# Supplementary material for: Evaluating Limits of Machine Learning-Assisted Raman Spectroscopy in Classification of Biological Samples
Source: ACS Omega. 2026 Jul 30;11(31):47122–34. doi: 10.1021/acsomega.6c06456 (PMC13470739; doi:10.1021/acsomega.6c06456)
Supplement: Supplementary file 1 [file ao6c06456_si_002.pdf]

Supporting Information

Evaluating Limits of Machine Learning-Assisted Raman Spectroscopy in Classification of Biological Samples

Authors:

Aman Yadav[1], Arlin Birkby[1], Noah Armstrong[1], Assame Arnob[1], Ming-Hsun Chou[2,3], Alma Fernandez[2,4], Aart J. Verhoef[2,4], Zhenhuan Yi[2], Siddhant Gulati[1], Siddhi Kotnis[1], Qing Sun[1,5], Katy C. Kao[6], Hung-Jen Wu[1,*]

Affiliation:

[1] Artie McFerrin Department of Chemical Engineering, Texas A&M University, College Station, Texas 77843, USA

[2] Institute for Quantum Science and Engineering, Texas A&M University, College Station, TX 77843, USA

[3] Department of Physics and Astronomy, Texas A&M University, College Station, Texas 77843, USA

[4] Department of Soil and Crop Sciences, Texas A&M University, College Station, TX 77843, USA

[5] Interdisciplinary Graduate Program in Genetics and Genomics, Texas A&M University, College Station, Texas 77843, USA

[6] Department of Chemical and Materials Engineering, San Jose State University, San Jose 95112-3613, California, USA

* Corresponding authors

Figure S1 Custom-assembled Raman microscope. 5
Figure S2 (a) Normalized Raman spectra for different compositions of Glyceryl trioctanoate (GTO) and Octanoic acid (OA) generated using a weighted average of pure GTO and OA. (b) 200–1000 cm^{-1}, (c) 1100–1500 cm^{-1}, and (d) 1600–1800 cm^{-1} are enlarged versions of 8
Figure S3 Scatter plot for similarity score for 90% GTO 10% OA compared to 100% GTO using Pair Distance (PD) for noise levels (σ) (a) 0.5 and (b) 15. As noise increases (σ = 15), intra-group similarity scores become substantially more spread out and overlap with inter-group scores, resulting in reduced classification accuracy. Red dots and error bars represent the mean and standard deviation of similarity scores for each group. 10
Figure S4 PCA loading plot highlighting the major wavenumbers contributing to PC1 for the GTO-OA mixture dataset. The PC1 loading spectrum shows prominent contributing bands at approximately 273, 910, 1414, 1661, and 1746 cm^{-1}, marked by vertical dashed lines. 14
Figure S5 SVM coefficient profiles for distinguishing GTO-OA mixtures from pure GTO. The SVM coefficient spectra show the wavenumber regions that contribute to the classification between pure GTO and mixtures containing 10%, 0.625%, and 0.0195% OA. Larger coefficient magnitudes indicate spectral regions with greater contribution to class separation, while the reduced coefficients at lower OA concentrations reflect decreasing spectral differences between the mixtures and pure GTO. 15
Figure S6 Row-wise normalized confusion matrix for multiclass classification of simulated mixtures, average standard deviation 1.67×10-4 (σ=0.5), using SVM-Linear. 16
Figure S7 Row-wise normalized confusion matrix for multiclass classification of simulated mixtures, average standard deviation 3.33×10-4 (σ=1), using SVM-Linear. 16
Figure S8 Row-wise normalized confusion matrix for multiclass classification of simulated mixtures, average standard deviation 6.64×10-4 (σ=2), using SVM-Linear. 17
Figure S9 Row-wise normalized confusion matrix for multiclass classification of simulated mixtures, average standard deviation 1.62×10-3 (σ=5), using SVM-Linear. 17
Figure S10 Row-wise normalized confusion matrix for multiclass classification of simulated mixtures, average standard deviation 2.23×10-3 (σ=7), using SVM-Linear. 18
Figure S11 Row-wise normalized confusion matrix for multiclass classification of simulated mixtures, average standard deviation 3.08×10-3 (σ=10), using SVM-Linear. 18
Figure S12 Row-wise normalized confusion matrix for multiclass classification of simulated mixtures, average standard deviation 4.33×10-3 (σ=15), using SVM-Linear. 19
Figure S13 Row-wise normalized confusion matrix for multiclass classification of simulated mixtures, average standard deviation 8.32×10-3 (σ=45), using SVM-Linear. 19
Figure S14 Row-wise normalized confusion matrix for multiclass classification of simulated mixtures, average standard deviation 9.14×10-3 (σ=60), using SVM-Linear. 20
Figure S15 Classification accuracy for different composition differences (a) Results for intra-day measurements and (b) Results for inter-day measurements. 21
Figure S16 Row-wise normalized confusion matrix for multiclass classification of intra-day data using Naïve Bayes Gaussian. 22
Figure S17 Row-wise normalized confusion matrix for multiclass classification of intra-day data using Decision Tree. 23
Figure S18 Row-wise normalized confusion matrix for multiclass classification of intra-day data using KNN-Euclidean. 23
Figure S19 Row-wise normalized confusion matrix for multiclass classification of intra-day data using Neural Network. 24

Figure S20 Row-wise normalized confusion matrix for multiclass classification of intra-day data using Convolutional Neural Network. 24
Figure S21 Row-wise normalized confusion matrix for multiclass classification of inter-day data using Naïve Bayes Gaussian. 25
Figure S22 Row-wise normalized confusion matrix for multiclass classification of inter-day data using Decision Tree. 25
Figure S23 Row-wise normalized confusion matrix for multiclass classification of inter-day data using KNN-Euclidean. 26
Figure S24 Row-wise normalized confusion matrix for multiclass classification of inter-day data using Neural Network. 26
Figure S25 Row-wise normalized confusion matrix for multiclass classification of inter-day data using Convolutional Neural Network. 27
Figure S26 Mean spectra and double standard deviations (shaded region) for E. coli, EBY100, L. lactis, L. reuteri, YAG01, YAG02, YAG03, YAG04, YAG05, YAG06, YAG07, YAG08, YAG09, YAG10, YAG17, YAG20, YAG22, YAG23, YAG28, and YLH2. 31
Figure S27 Binary classification results for all the cells compared to YLH2 using Naïve Bayes Gaussian. 32
Figure S28 Binary classification results for all the cells compared to YLH2 using Decision Tree. 32
Figure S29 Binary classification results for all the cells compared to YLH2 using KNN-Euclidean. 33
Figure S30 Binary classification results for all the cells compared to YLH2 using Neural Network. 33
Figure S31 Binary classification results for all the cells compared to YLH2 using Convolutional Neural Network. 34
Figure S32 Row-wise normalized confusion matrix for multiclass classification of cells using Naïve Bayes Gaussian (Overall accuracy: 52.08%). 35
Figure S33 Row-wise normalized confusion matrix for multiclass classification of cells using a Decision Tree (Overall accuracy: 54.79%). 36
Figure S34 Row-wise normalized confusion matrix for multiclass classification of cells using KNN-Euclidean (Overall accuracy: 55.76%). 37
Figure S35 Row-wise normalized confusion matrix for multiclass classification of cells using a Neural network (Overall accuracy- 62.29%). 38
Figure S36 Row-wise normalized confusion matrix for multiclass classification of cells using a Convolutional Neural Network (Overall accuracy- 68.75%). 39
Figure S37 PCA loading plot highlighting the major wavenumbers contributing to PC1 for the cells dataset. The PC1 loading spectrum shows prominent contributing bands at approximately 1006, 1157, 1344, 1448, and 1524 cm^{-1}, marked by vertical dashed lines. 40

Table S1 Raman peak assignments for Glyceryl trioctanoate vibrational modes. 5
Table S2 Raman peak assignments for Octanoic Acid vibrational modes. 6
Table S3 Composition (vol%) of mixtures prepared using the weighted average of the spectra from pure Glyceryl Trioctanoate (GTO) and pure Octanoic Acid (OA). 7
Table S4 Similarity score for spectra with different binary mixtures of GTO and OA compared to pure GTO (100%) using the two similarity metrics for average standard deviation 1.67×10-4 (σ=0.5). Green indicates high similarity, while red represents dissimilarity. 9
Table S5 Average standard deviation for each noise level for the simulated spectra. 10
Table S6 Classification accuracy scores for different composition differences with varying average standard deviation values using Naive Bayes Gaussian. 11
Table S7 Classification accuracy scores for different composition differences with varying average standard deviation values using a Decision Tree. 11
Table S8 Classification accuracy scores for different composition differences with varying average standard deviation values using SVM. 12
Table S9 Classification accuracy scores for different composition differences with varying average standard deviation values using KNN-Euclidean. 12
Table S10 Classification accuracy score for different composition differences with varying average standard deviation values using a Neural Network. 13
Table S11 Classification accuracy scores for different composition differences with varying average standard deviation values using a Convolutional Neural Network. 13
Table S12 Label details for multiclass classification confusion matrices. 15
Table S13 Average standard deviation value for inter-day and intra-samples for all the mixtures. 20
Table S14 Comparison of classification accuracy using a Linear Support Vector Machine (SVM-Linear) for simulated versus experimental intra-day sample data. 21
Table S15 Comparison of classification accuracy using a Linear Support Vector Machine (SVM-Linear) for simulated versus experimental inter-day sample data. 22
Table S16 Strain and mutation label details of microorganisms used in the study. 28
Table S17 Average standard deviation of Raman spectra in each microorganism. 29
Table S18 Similarity scores for spectra of various mutated yeast strains and other microorganisms compared to the industrial strain YLH2. Green indicates high similarity, while red represents dissimilarity 30
Table S19 Raman peak assignments for mutated yeast strains. 40

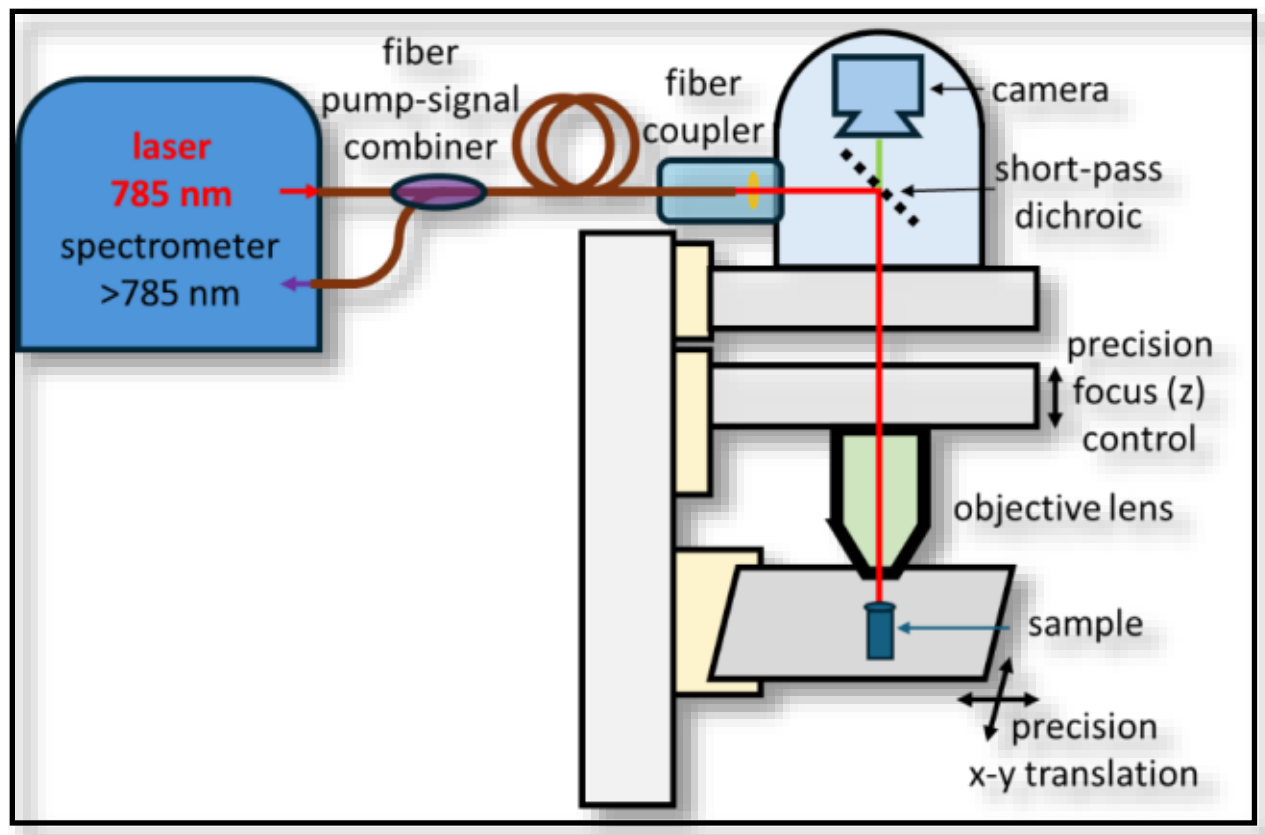

Figure S1 Custom-assembled Raman microscope.

**Wavenumber (cm^{-1})**	**Vibration modes**	**Reference**
~216	Low-frequency CH_3-CH_2 , CH_2-CH_2 torsion	[1]
~404	Chain expansion n-Alkanes	[1]
~606	CCC deformation	[1]
~843	C-C-C stretching mode	[1]
~896	Methyl Terminal rocking	[1]
~922	C–C stretching/ester-associated vibrations	[1]
~1034	C-C stretching mode	[2]
~1076	C-C stretching mode	[2]
~1119	C-C stretching mode	[2]
~1306	CH_2 twisting	[2]
~1444	CH_2 scissoring mode (1445 cm^{-1})	[3],3
~1747	C=O ester stretching vibrations	[3], [4]

Table S1 Raman peak assignments for Glyceryl trioctanoate vibrational modes.

**Wavenumber (cm^{-1})**	**Vibration modes**	**References**
~271	CH3-CH2 , CH2-CH2 torsion	[1]
~321	CCC deformation	[1]
~403	Chain expansion n-Alkanes	[1]
~732	CH2 rocking	[1]
~793	C–C skeletal vibration	[1]
~845	C-C-C stretching mode	[1]
~899	Methyl Terminal rocking	[1]
~1035	C-C stretching mode	[2]
~1080	C-C stretching mode	[2]
~1119	C-C stretching mode	[2]
~1177	C-C stretching mode	[2]
~1306	CH_2 twisting	[2]
~1444	CH_2 scissoring mode (1445 cm^{-1})	[3], [4])
~1661	Tentative stretching vibrations of carboxyl and carbonyl C=O	[3], [4]

Table S2 Raman peak assignments for Octanoic Acid vibrational modes.

GTO (Vol %)	OA (Vol %)
25	75
50	50
75	25
90	10
95	5
97.5	2.5
98.75	1.25
99.375	0.625
99.6875	0.3125
99.8438	0.1562
99.9219	0.0781
99.9609	0.0391
99.9805	0.0195

Table S3 Composition (vol%) of mixtures prepared using the weighted average of the spectra from pure Glyceryl Trioctanoate (GTO) and pure Octanoic Acid (OA).

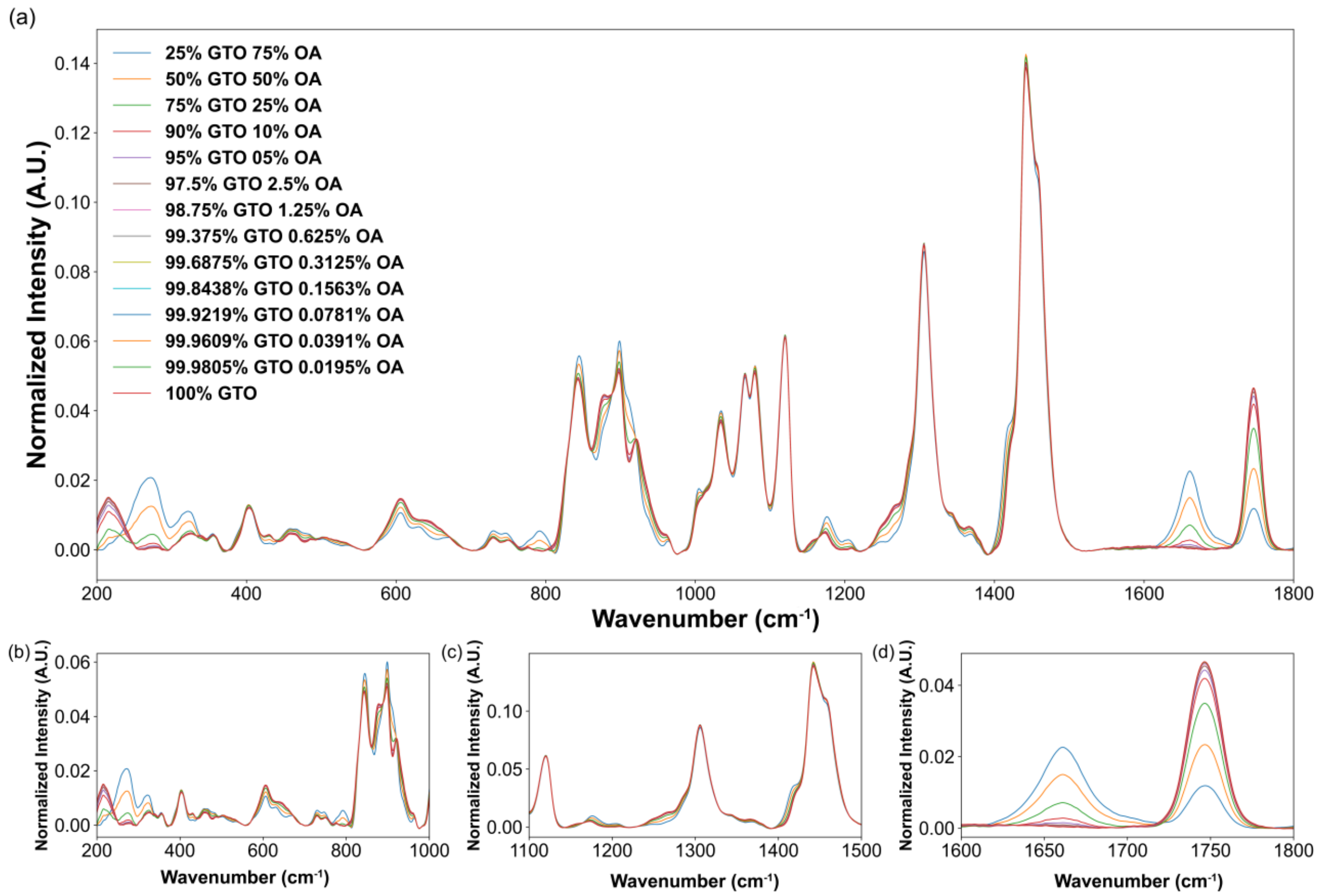

Figure S2 (a) Normalized Raman spectra for different compositions of Glyceryl trioctanoate (GTO) and Octanoic acid (OA) generated using a weighted average of pure GTO and OA. (b) 200–1000 cm^{-1}, (c) 1100–1500 cm^{-1}, and (d) 1600–1800 cm^{-1} are enlarged versions of

Mixture Compositions	Similarity Metrics	
	PC	PD
**25% GTO 75% OA**	0.952229	0.034831
**50% GTO 50% OA**	0.97898	0.02335
**75% GTO 25% OA**	0.994218	0.011825
**90% GTO 10% OA**	0.99897	0.004919
**95% GTO 5% OA**	0.999679	0.002752
**97.5% GTO 2.5% OA**	0.999872	0.00168
**98.75% GTO 1.25% OA**	0.999923	0.001121
**99.375% GTO 0.625% OA**	0.999936	0.000899
**99.6875% GTO 0.3125% OA**	0.999939	0.00085
**99.8438% GTO 0.1563% OA**	0.99994	0.000839
**99.9219% GTO 0.0781% OA**	0.99994	0.000837
**99.9609% GTO 0.0391% OA**	0.99994	0.000836
**99.9805% GTO 0.0195% OA**	0.99994	0.000836

Table S4 Similarity score for spectra with different binary mixtures of GTO and OA compared to pure GTO (100%) using the two similarity metrics for average standard deviation 1.67×10-4 (σ=0.5). Green indicates high similarity, while red represents dissimilarity.

Noise Levels (σ)	Average Standard Deviation
**0.5**	1.67×10^{-4}
**1**	3.33×10^{-4}
**2**	6.64×10^{-4}
**5**	1.62×10^{-3}
**7**	2.23×10^{-3}
**10**	3.08×10^{-3}
**15**	4.33×10^{-3}
**45**	8.32×10^{-3}
**60**	9.14×10^{-3}

Table S5 Average standard deviation for each noise level for the simulated spectra.

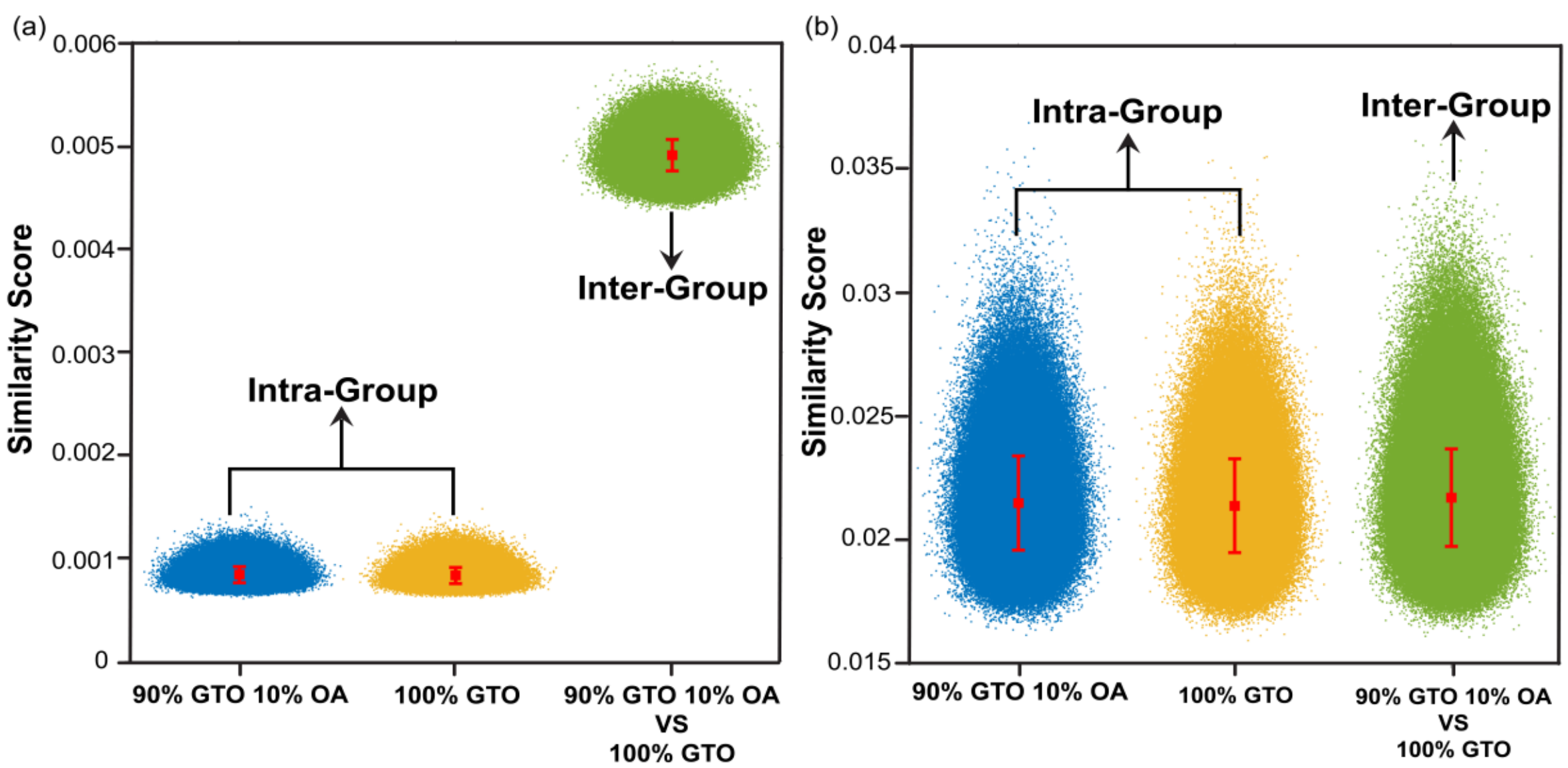

Figure S3 Scatter plot for similarity score for 90% GTO 10% OA compared to 100% GTO using Pair Distance (PD) for noise levels (σ) (a) 0.5 and (b) 15. As noise increases (σ = 15), intra-group similarity scores become substantially more spread out and overlap with inter-group scores, resulting in reduced classification accuracy. Red dots and error bars represent the mean and standard deviation of similarity scores for each group.

Mixture Compositions	Average standard deviation								
	1.67×10^{-4} (σ=0.5)	3.33×10^{-4} (σ=1)	6.64×10^{-4} (σ=2)	1.62×10^{-3} (σ=5)	2.23×10^{-3} (σ=7)	3.08×10^{-3} (σ=10)	4.33×10^{-3} (σ=15)	8.32×10^{-3} (σ=45)	9.14×10^{-3} (σ=60)
25% GTO 75% OA	100%	100%	100%	100%	100%	100%	100%	100%	100%
50% GTO 50% OA	100%	100%	100%	100%	100%	100%	100%	100%	99.2%
75% GTO 25% OA	100%	100%	100%	100%	100%	100%	100%	97.65%	93.95%
90% GTO 10% OA	100%	100%	100%	100%	100%	99.75%	96.85%	70%	59.5%
95% GTO 5% OA	100%	100%	100%	99.4%	97.45%	96.7%	85.4%	55.5%	53.15%
97.5% GTO 2.5% OA	100%	100%	99.95%	95.7%	88.15%	76.65%	59.5%	53.55%	53.4%
98.75% GTO 1.25% OA	100%	99.9%	96.7%	74.85%	64.05%	58.25%	53.8%	52.55%	52.3%
99.375% GTO 0.625% OA	99.95%	96.4%	83.7%	57.05%	55.1%	54.45%	53.9%	52.05%	50.9%
99.6875% GTO 0.3125% OA	95.6%	81.95%	61.45%	52.1%	52.05%	52.4%	52%	52.7%	53.15%
99.8438% GTO 0.1563% OA	82.25%	58.55%	53.95%	52.4%	53%	50.85%	51.65%	52.5%	51.15%
99.9219% GTO 0.0781% OA	62.75%	55.05%	52.15%	51.5%	52.9%	51.85%	52.65%	52.3%	52.55%
99.9609% GTO 0.0391% OA	53.45%	53.6%	50.35%	51.8%	50.9%	51.5%	52.25%	51.45%	52.85%
99.9805% GTO 0.0195% OA	51.6%	52.45%	51.4%	51.15%	52.95%	51.7%	52.4%	47.6%	51.75%

Table S6 Classification accuracy scores for different composition differences with varying average standard deviation values using Naive Bayes Gaussian.

Mixture Compositions	Average standard deviation								
	1.67×10^{-4} (σ=0.5)	3.33×10^{-4} (σ=1)	6.64×10^{-4} (σ=2)	1.62×10^{-3} (σ=5)	2.23×10^{-3} (σ=7)	3.08×10^{-3} (σ=10)	4.33×10^{-3} (σ=15)	8.32×10^{-3} (σ=45)	9.14×10^{-3} (σ=60)
25% GTO 75% OA	100%	100%	100%	100%	100%	100%	100%	100%	100%
50% GTO 50% OA	100%	100%	100%	100%	100%	100%	100%	100%	99.15%
75% GTO 25% OA	100%	100%	100%	100%	100%	100%	100%	84.05%	78.05%
90% GTO 10% OA	100%	100%	100%	100%	100%	99.7%	94.45%	61.3%	53.95%
95% GTO 5% OA	100%	100%	100%	99.65%	95.55%	86.05%	74.1%	52.7%	52.55%
97.5% GTO 2.5% OA	100%	100%	99.85%	84.25%	73.75%	64.45%	54.4%	51.9%	52.5%
98.75% GTO 1.25% OA	100%	99.75%	92.15%	61.3%	55.65%	53.95%	52.05%	52.3%	51.6%
99.375% GTO 0.625% OA	99.9%	94.5%	68.75%	54.6%	52.9%	52.75%	53.8%	52.85%	52.65%
99.6875% GTO 0.3125% OA	91.6%	68.8%	53.8%	51.95%	52.3%	52.15%	53.35%	52.65%	52.75%
99.8438% GTO 0.1563% OA	71.2%	54.55%	52.25%	52.1%	53.65%	51.4%	51.9%	52.15%	52.2%
99.9219% GTO 0.0781% OA	55.05%	51.9%	51.85%	51.7%	51.7%	52.3%	51.75%	52.65%	52.2%
99.9609% GTO 0.0391% OA	51.55%	53.35%	51.4%	52.9%	51.45%	51.75%	52.45%	52.25%	53.05%
99.9805% GTO 0.0195% OA	52.65%	52.15%	52.6%	53.05%	52.1%	51.75%	51.55%	52.45%	52.85%

Table S7 Classification accuracy scores for different composition differences with varying average standard deviation values using a Decision Tree.

Mixture Compositions	Average standard deviation								
	1.67×10^{-4} (σ=0.5)	3.33×10^{-4} (σ=1)	6.64×10^{-4} (σ=2)	1.62×10^{-3} (σ=5)	2.23×10^{-3} (σ=7)	3.08×10^{-3} (σ=10)	4.33×10^{-3} (σ=15)	8.32×10^{-3} (σ=45)	9.14×10^{-3} (σ=60)
25% GTO 75% OA	100%	100%	100%	100%	100%	100%	100%	100%	100%
50% GTO 50% OA	100%	100%	100%	100%	100%	100%	100%	100%	100%
75% GTO 25% OA	100%	100%	100%	100%	100%	100%	100%	98.6%	93.85%
90% GTO 10% OA	100%	100%	100%	100%	100%	100%	99.65%	71%	60.4%
95% GTO 5% OA	100%	100%	100%	99.95%	99.8%	97.85%	86.75%	56.7%	53.35%
97.5% GTO 2.5% OA	100%	100%	100%	97.3%	88.65%	77.5%	59.4%	52.8%	52.5%
98.75% GTO 1.25% OA	100%	100%	99.1%	75.75%	65.5%	59.15%	54.8%	53.15%	52.95%
99.375% GTO 0.625% OA	100%	98.95%	83.95%	56.9%	54.45%	55.5%	54.45%	52.4%	50.4%
99.6875% GTO 0.3125%OA	99%	82.75%	62.4%	53.8%	51%	52.2%	52%	51.8%	53.3%
99.8438% GTO 0.1563%OA	83.2%	58.5%	53.7%	53.25%	50.1%	51.5%	51.05%	53.1%	52.55%
99.9219% GTO 0.0781% OA	62.95%	55.15%	52.55%	52%	52.65%	52.4%	52.85%	53.35%	51.75%
99.9609% GTO 0.0391% OA	53.95%	54.1%	49.85%	53.35%	51.55%	52.4%	51.95%	50.9%	53.35%
99.9805% GTO 0.0195% OA	52%	52.25%	50.9%	50.95%	52.9%	52.2%	52.8%	47.6%	52.85%

Table S8 Classification accuracy scores for different composition differences with varying average standard deviation values using SVM.

Mixture Compositions	Average standard deviation								
	1.67×10^{-4} (σ=0.5)	3.33×10^{-4} (σ=1)	6.64×10^{-4} (σ=2)	1.62×10^{-3} (σ=5)	2.23×10^{-3} (σ=7)	3.08×10^{-3} (σ=10)	4.33×10^{-3} (σ=15)	8.32×10^{-3} (σ=45)	9.14×10^{-3} (σ=60)
25% GTO 75% OA	100%	100%	100%	100%	100%	100%	100%	100%	100%
50% GTO 50% OA	100%	100%	100%	100%	100%	100%	100%	100%	99.65%
75% GTO 25% OA	100%	100%	100%	100%	100%	100%	100%	95.1%	86.3%
90% GTO 10% OA	100%	100%	100%	100%	100%	99.9%	97.35%	59.2%	53.3%
95% GTO 5% OA	100%	100%	100%	99.9%	98.15%	92.9%	73.15%	53%	53.3%
97.5% GTO 2.5% OA	100%	100%	100%	90.8%	79.15%	64.9%	54.2%	53.65%	51.45%
98.75% GTO 1.25% OA	100%	99.95%	95.6%	62%	56.05%	53.75%	53.15%	52.7%	52.65%
99.375% GTO 0.625% OA	99.95%	95.3%	71.15%	52.55%	52.95%	52.45%	53.35%	52.6%	51.05%
99.6875% GTO 0.3125% OA	94.6%	68.7%	55.6%	52.85%	51.8%	52.9%	52.25%	52.2%	51.2%
99.8438% GTO 0.1563% OA	70.35%	54.9%	52.15%	52.4%	51.9%	51.8%	53.05%	53.75%	52.05%
99.9219% GTO 0.0781% OA	55.05%	53.5%	52.4%	51.25%	52.65%	51%	52%	52.75%	52.05%
99.9609% GTO 0.0391% OA	53.65%	52.2%	52.1%	51%	52.35%	51.85%	51.8%	54.85%	51.8%
99.9805% GTO 0.0195% OA	52.4%	52.75%	51.4%	52.9%	52.75%	52.05%	51.7%	51.6%	51.45%

Table S9 Classification accuracy scores for different composition differences with varying average standard deviation values using KNN-Euclidean.

Mixture Compositions	Average standard deviation								
	1.67×10^{-4} (σ=0.5)	3.33×10^{-4} (σ=1)	6.64×10^{-4} (σ=2)	1.62×10^{-3} (σ=5)	2.23×10^{-3} (σ=7)	3.08×10^{-3} (σ=10)	4.33×10^{-3} (σ=15)	8.32×10^{-3} (σ=45)	9.14×10^{-3} (σ=60)
25% GTO 75% OA	100%	100%	100%	100%	100%	100%	100%	100%	100%
50% GTO 50% OA	100%	100%	100%	100%	100%	100%	100%	100%	100%
75% GTO 25% OA	100%	100%	100%	100%	100%	100%	100%	98.65%	93.95%
90% GTO 10% OA	100%	100%	100%	100%	100%	100%	99.8%	70.8%	60.3%
95% GTO 5% OA	100%	100%	100%	99.95%	99.85%	97.9%	87.1%	55.85%	52.8%
97.5% GTO 2.5% OA	100%	100%	100%	97.35%	88.85%	76.45%	59%	54.55%	52.75%
98.75% GTO 1.25% OA	100%	100%	99%	75.35%	64.7%	58.25%	54.65%	52.6%	52.45%
99.375% GTO 0.625% OA	100%	99.05%	83.35%	56%	53.95%	54.45%	53.35%	52.85%	51.4%
99.6875% GTO 0.3125% OA	98.9%	82.5%	61.4%	52.6%	52.15%	52.2%	52%	51.85%	53.3%
99.8438% GTO 0.1563% OA	82.95%	59.75%	54.05%	51.55%	52.65%	52.8%	52.25%	53.25%	52.6%
99.9219% GTO 0.0781% OA	63.7%	54.1%	51.75%	51.7%	52.35%	53.35%	54.4%	52.85%	52.1%
99.9609% GTO 0.0391% OA	55.15%	54.55%	51.2%	53%	52%	51.75%	52.45%	53.1%	53.2%
99.9805% GTO 0.0195% OA	52.75%	52.45%	51.85%	52.8%	53.35%	52.05%	53%	49.6%	53.3%

Table S10 Classification accuracy score for different composition differences with varying average standard deviation values using a Neural Network.

Mixture Compositions	Average standard deviation								
	1.67×10^{-4} (σ=0.5)	3.33×10^{-4} (σ=1)	6.64×10^{-4} (σ=2)	1.62×10^{-3} (σ=5)	2.23×10^{-3} (σ=7)	3.08×10^{-3} (σ=10)	4.33×10^{-3} (σ=15)	8.32×10^{-3} (σ=45)	9.14×10^{-3} (σ=60)
25% GTO 75% OA	100%	100%	100%	100%	100%	100%	100%	100%	100%
50% GTO 50% OA	100%	100%	100%	100%	100%	100%	100%	100%	100%
75% GTO 25% OA	100%	100%	100%	100%	100%	100%	100%	99.2%	95.9%
90% GTO 10% OA	100%	100%	100%	100%	100%	100%	100%	80.45%	73.15%
95% GTO 5% OA	100%	100%	100%	100%	100%	98.6%	92.3%	67.1%	58.7%
97.5% GTO 2.5% OA	100%	100%	100%	98.45%	93.45%	84.6%	73.9%	56.5%	54.6%
98.75% GTO 1.25% OA	100%	100%	99.7%	84.8%	75.3%	67.7%	59.65%	55.7%	55.05%
99.375% GTO 0.625% OA	100%	99.25%	89.8%	65.2%	62.8%	58.25%	56%	55.3%	53.85%
99.6875% GTO 0.3125% OA	98.9%	89.6%	72%	56.15%	55%	53.45%	53.8%	54%	54.75%
99.8438% GTO 0.1563% OA	89.55%	73%	58.75%	52.7%	54.1%	53.5%	53.1%	54.6%	53.25%
99.9219% GTO 0.0781% OA	72.3%	56.3%	55.15%	53.7%	53.55%	54.75%	53.4%	54.5%	55.35%
99.9609% GTO 0.0391% OA	57%	53.2%	52.85%	52.35%	52.55%	53.5%	53.25%	54.8%	53.45%
99.9805% GTO 0.0195% OA	54.4%	52.55%	53.2%	52.55%	53.9%	53.5%	53.5%	53.1%	54.05%

Table S11 Classification accuracy scores for different composition differences with varying average standard deviation values using a Convolutional Neural Network.

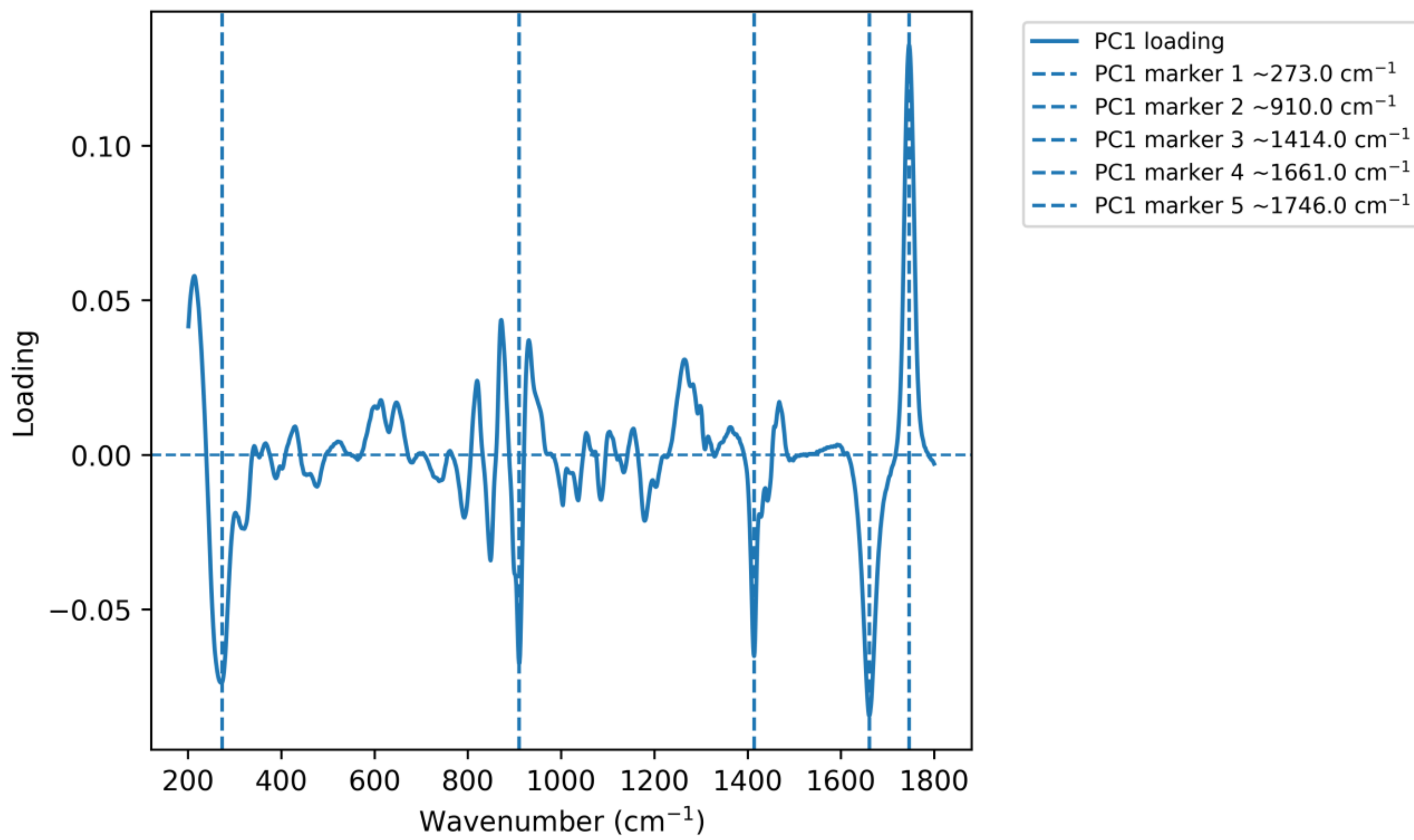

Figure S4 PCA loading plot highlighting the major wavenumbers contributing to PC1 for the GTO-OA mixture dataset. The PC1 loading spectrum shows prominent contributing bands at approximately 273, 910, 1414, 1661, and 1746 cm^{-1}, marked by vertical dashed lines.

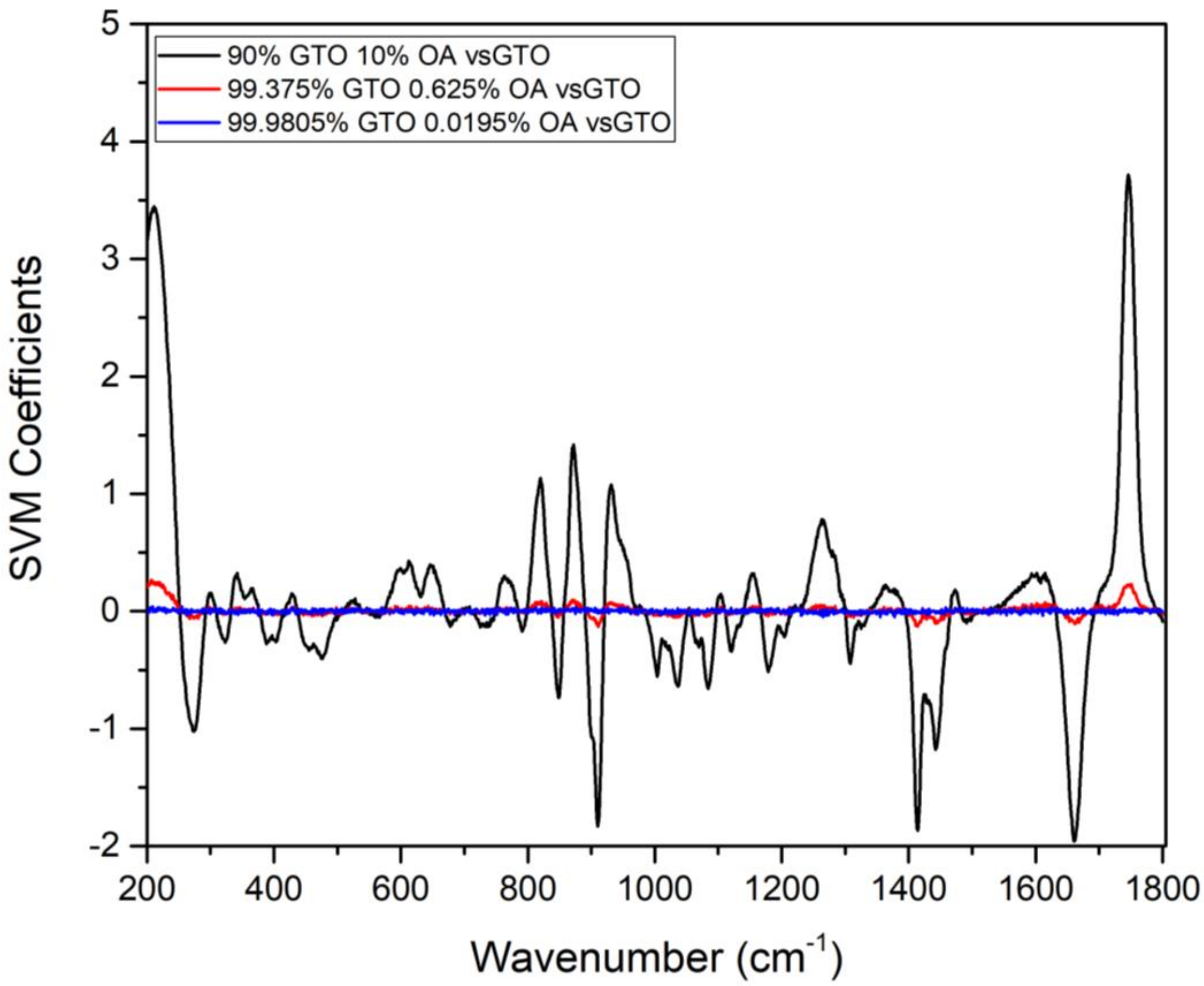

Figure S5 SVM coefficient profiles for distinguishing GTO-OA mixtures from pure GTO. The SVM coefficient spectra show the wavenumber regions that contribute to the classification between pure GTO and mixtures containing 10%, 0.625%, and 0.0195% OA. Larger coefficient magnitudes indicate spectral regions with greater contribution to class separation, while the reduced coefficients at lower OA concentrations reflect decreasing spectral differences between the mixtures and pure GTO.

**Mixture Compositions**	**Labels**
25% GTO 75% OA	25% GTO
50% GTO 50% OA	50% GTO
75% GTO 25% OA	75% GTO
90% GTO 10% OA	90% GTO
95% GTO 5% OA	95% GTO
97.5% GTO 2.5% OA	97.5% GTO
98.75% GTO 1.25% OA	98.75% GTO
99.375% GTO 0.625% OA	99.375% GTO
99.6875% GTO 0.3125% OA	99.6875% GTO
99.8438% GTO 0.1563% OA	99.8438% GTO
99.9219% GTO 0.0781% OA	99.9219% GTO
99.9609% GTO 0.0391% OA	99.9609% GTO
99.9805% GTO 0.0195% OA	99.9805% GTO
100% GTO	100% GTO

Table S12 Label details for multiclass classification confusion matrices.

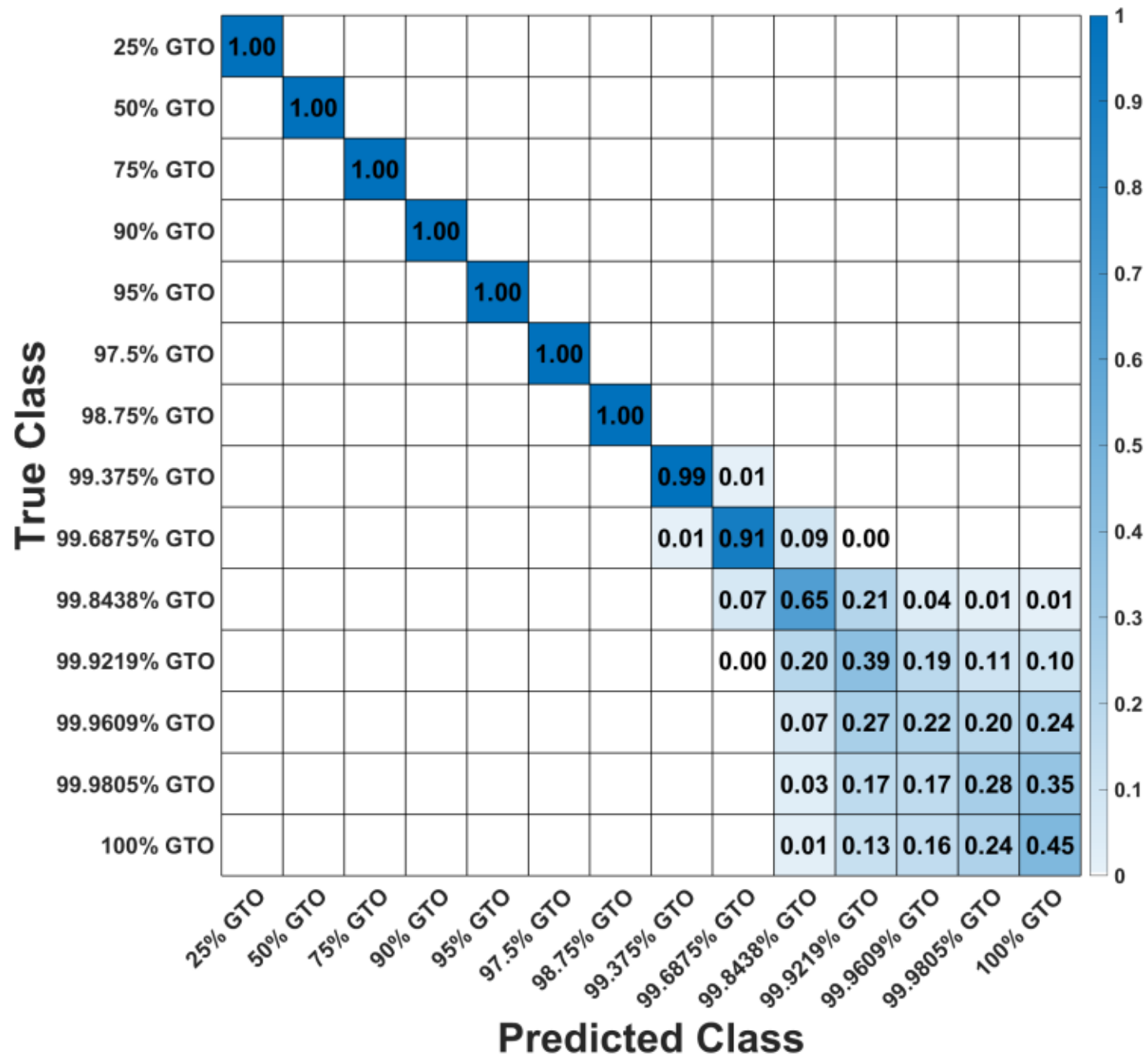

Figure S6 Row-wise normalized confusion matrix for multiclass classification of simulated mixtures, average standard deviation 1.67×10-4 (σ=0.5), using SVM-Linear.

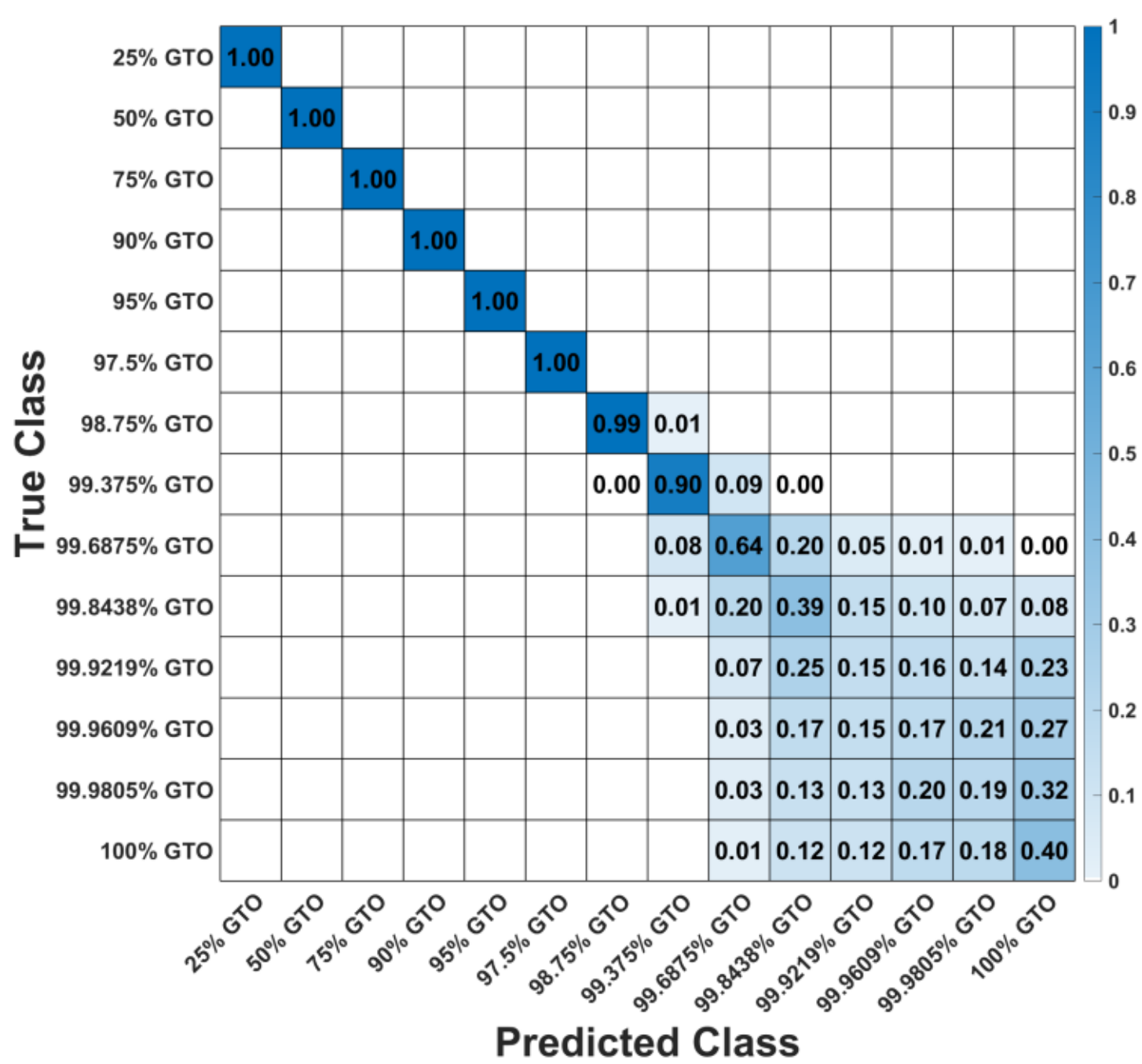

Figure S7 Row-wise normalized confusion matrix for multiclass classification of simulated mixtures, average standard deviation 3.33×10-4 (σ=1), using SVM-Linear.

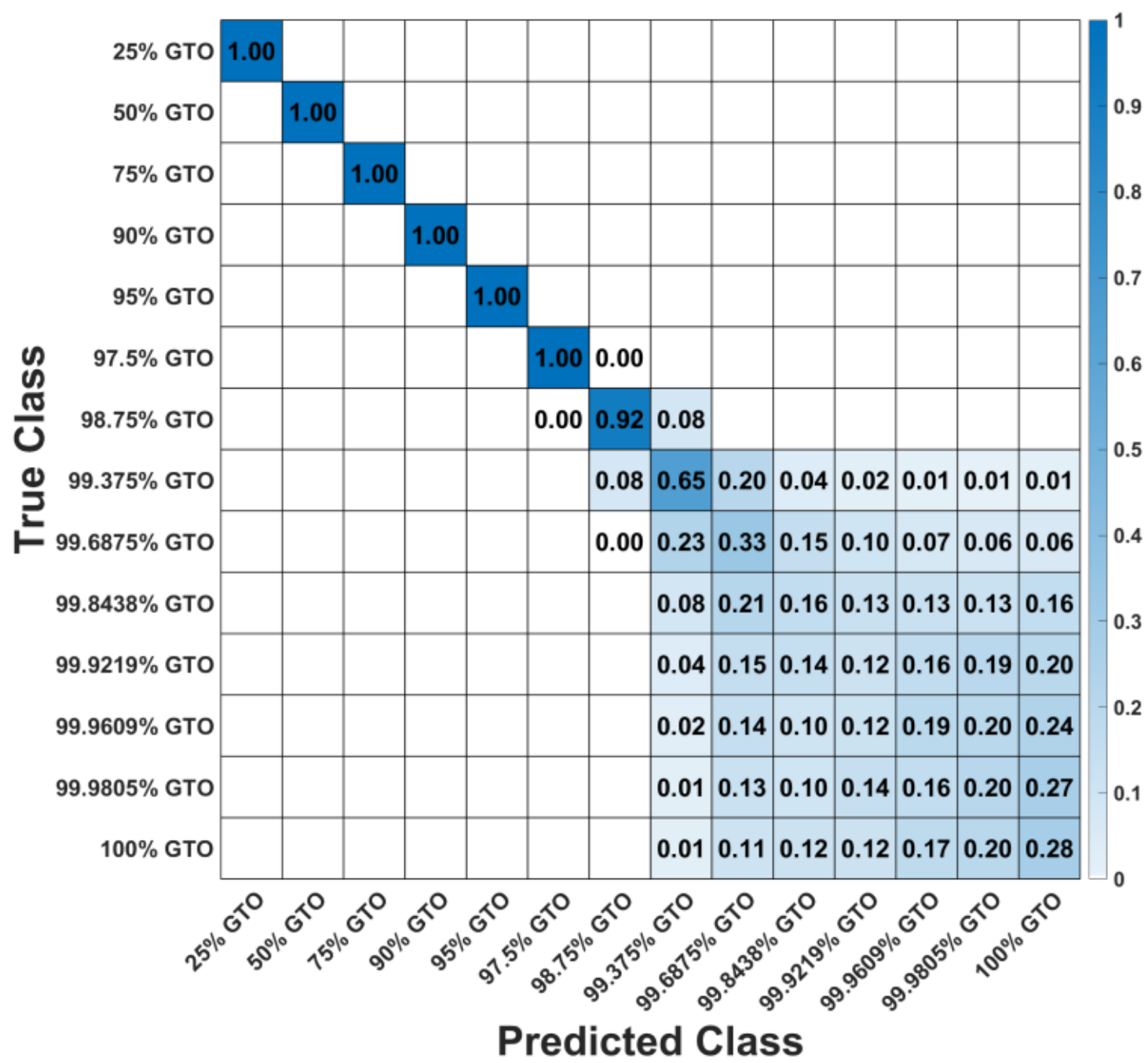

Figure S8 Row-wise normalized confusion matrix for multiclass classification of simulated mixtures, average standard deviation 6.64×10-4 (σ=2), using SVM-Linear.

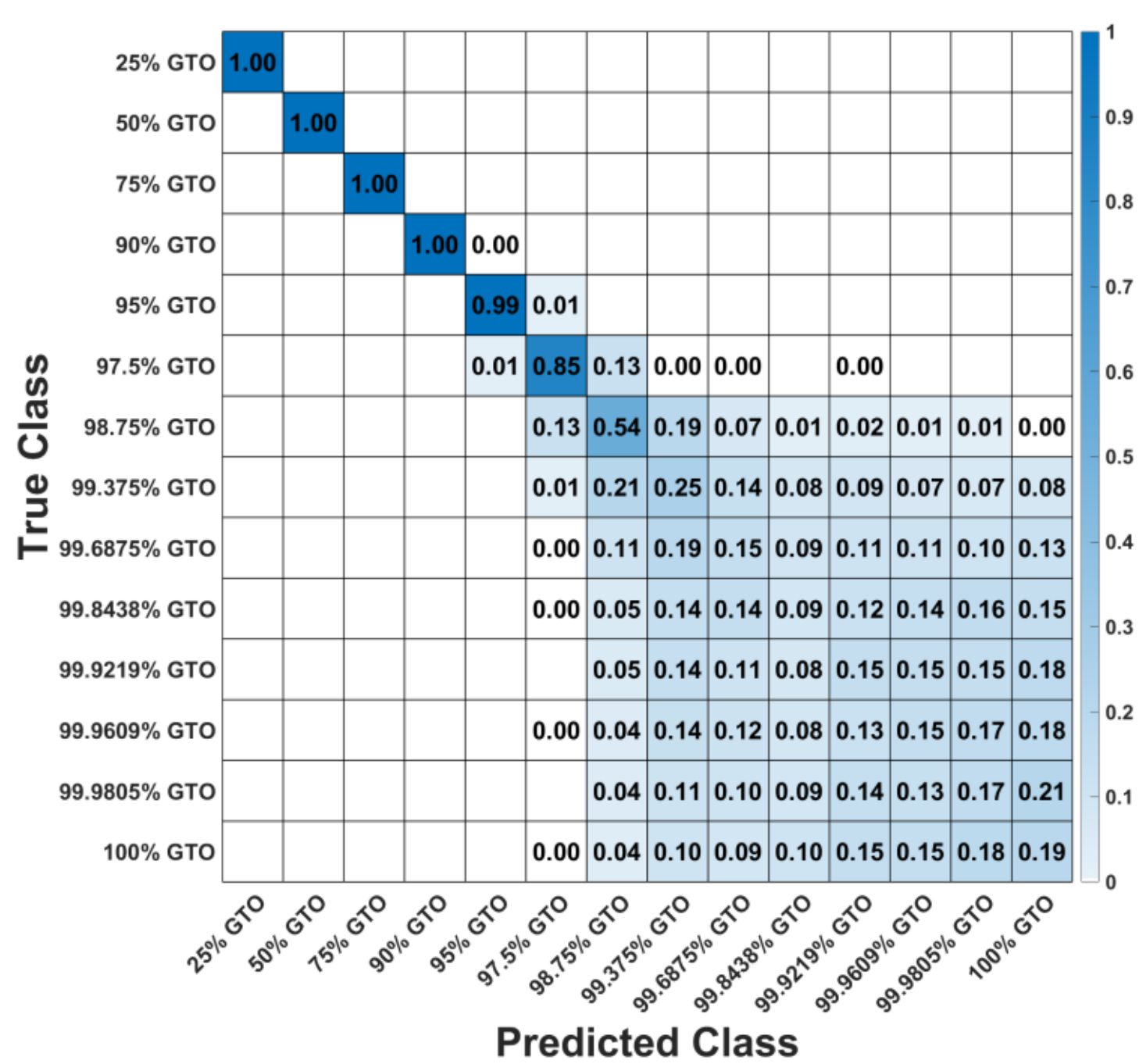

Figure S9 Row-wise normalized confusion matrix for multiclass classification of simulated mixtures, average standard deviation 1.62×10-3 (σ=5), using SVM-Linear.

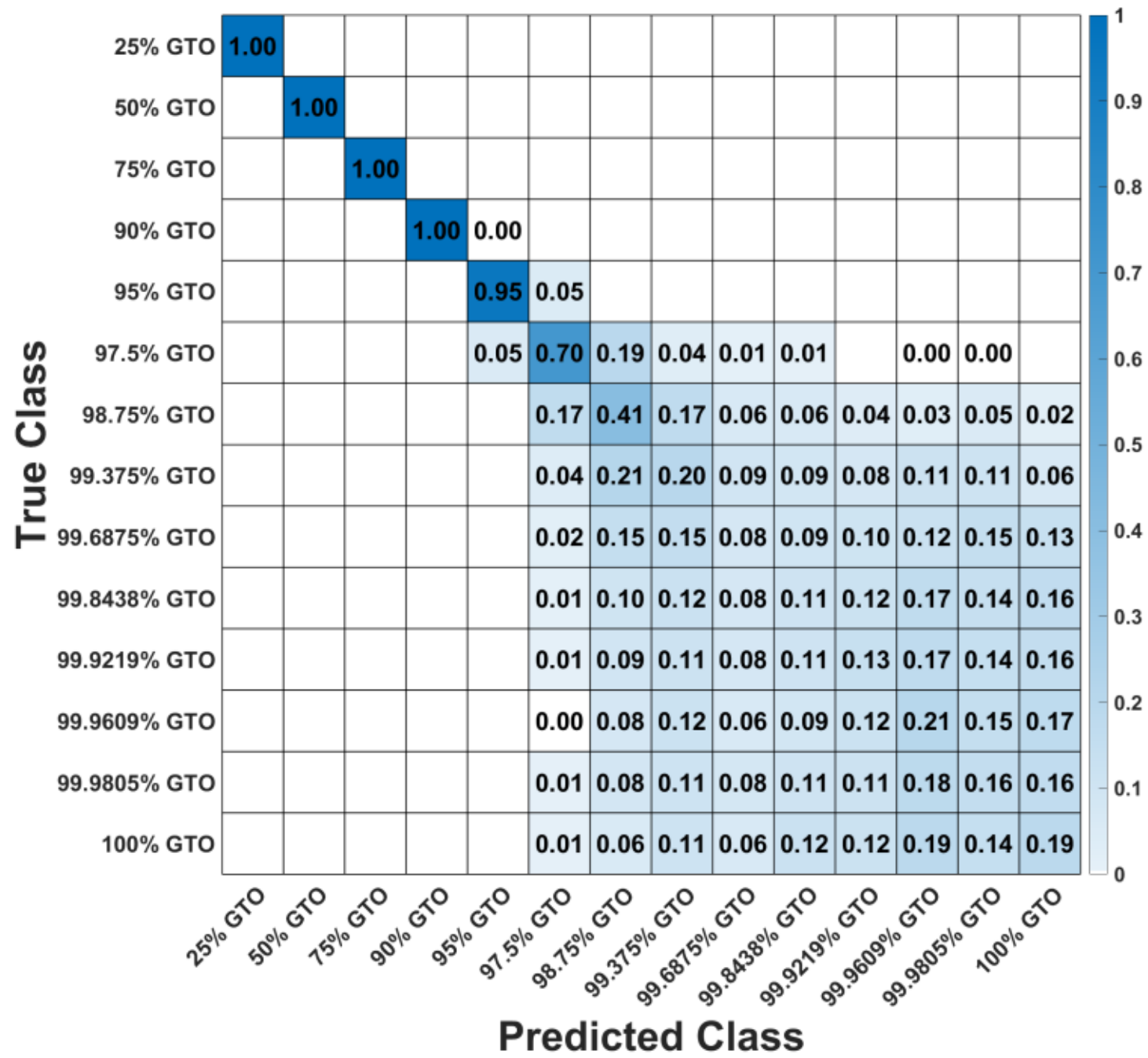

Figure S10 Row-wise normalized confusion matrix for multiclass classification of simulated mixtures, average standard deviation 2.23×10-3 (σ=7), using SVM-Linear.

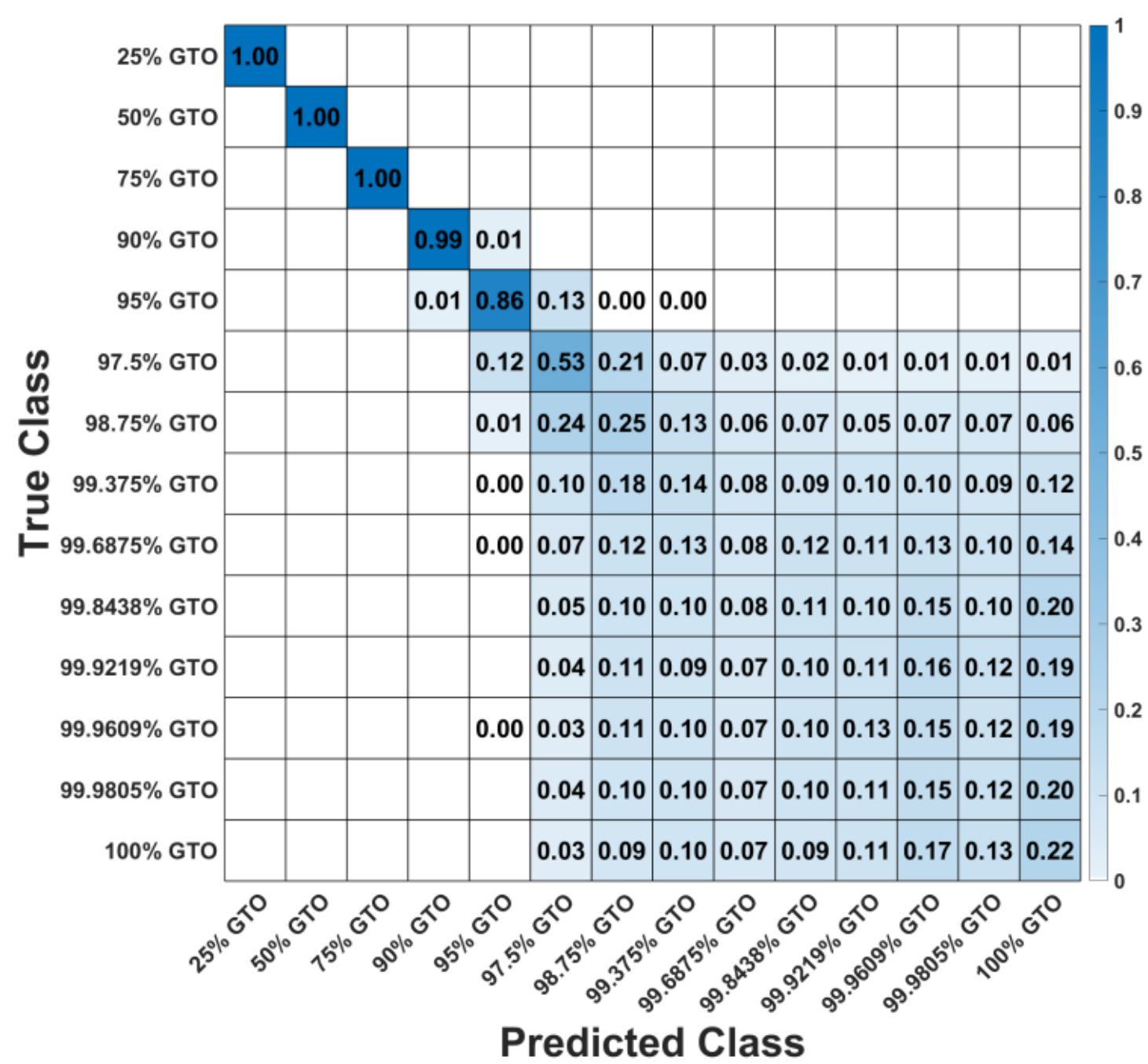

Figure S11 Row-wise normalized confusion matrix for multiclass classification of simulated mixtures, average standard deviation 3.08×10-3 (σ=10), using SVM-Linear.

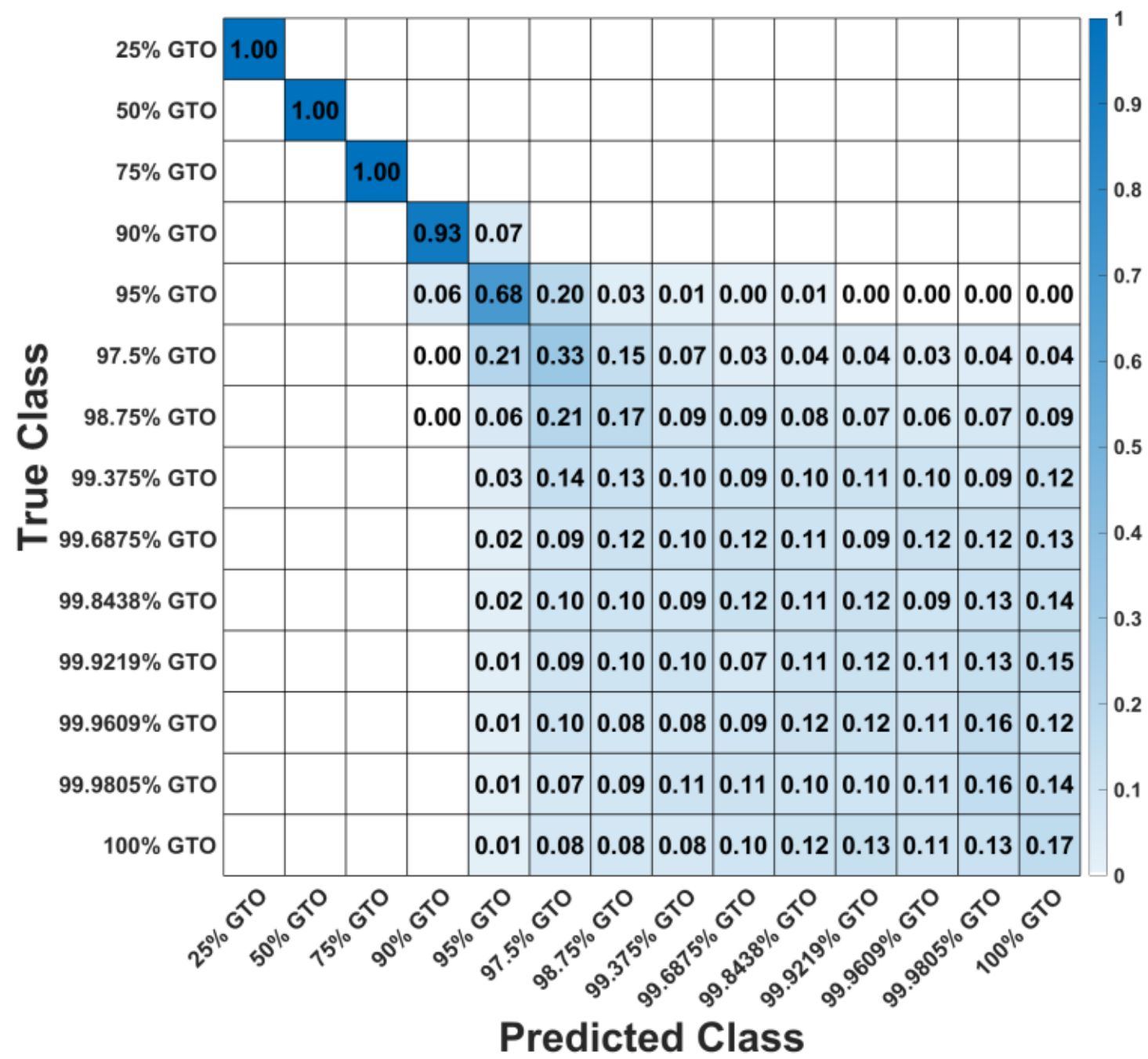

Figure S12 Row-wise normalized confusion matrix for multiclass classification of simulated mixtures, average standard deviation 4.33×10-3 (σ=15), using SVM-Linear.

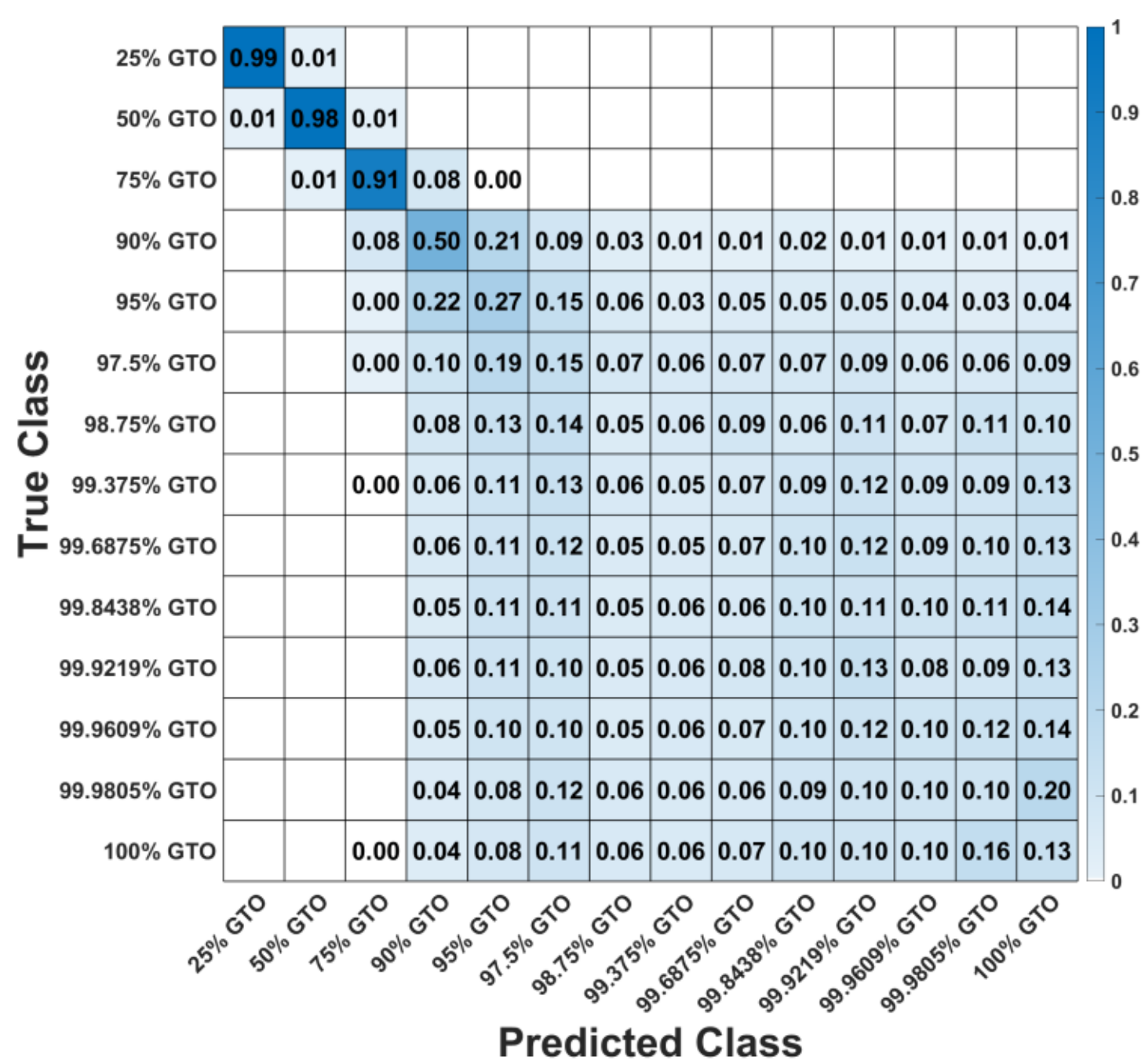

Figure S13 Row-wise normalized confusion matrix for multiclass classification of simulated mixtures, average standard deviation 8.32×10-3 (σ=45), using SVM-Linear.

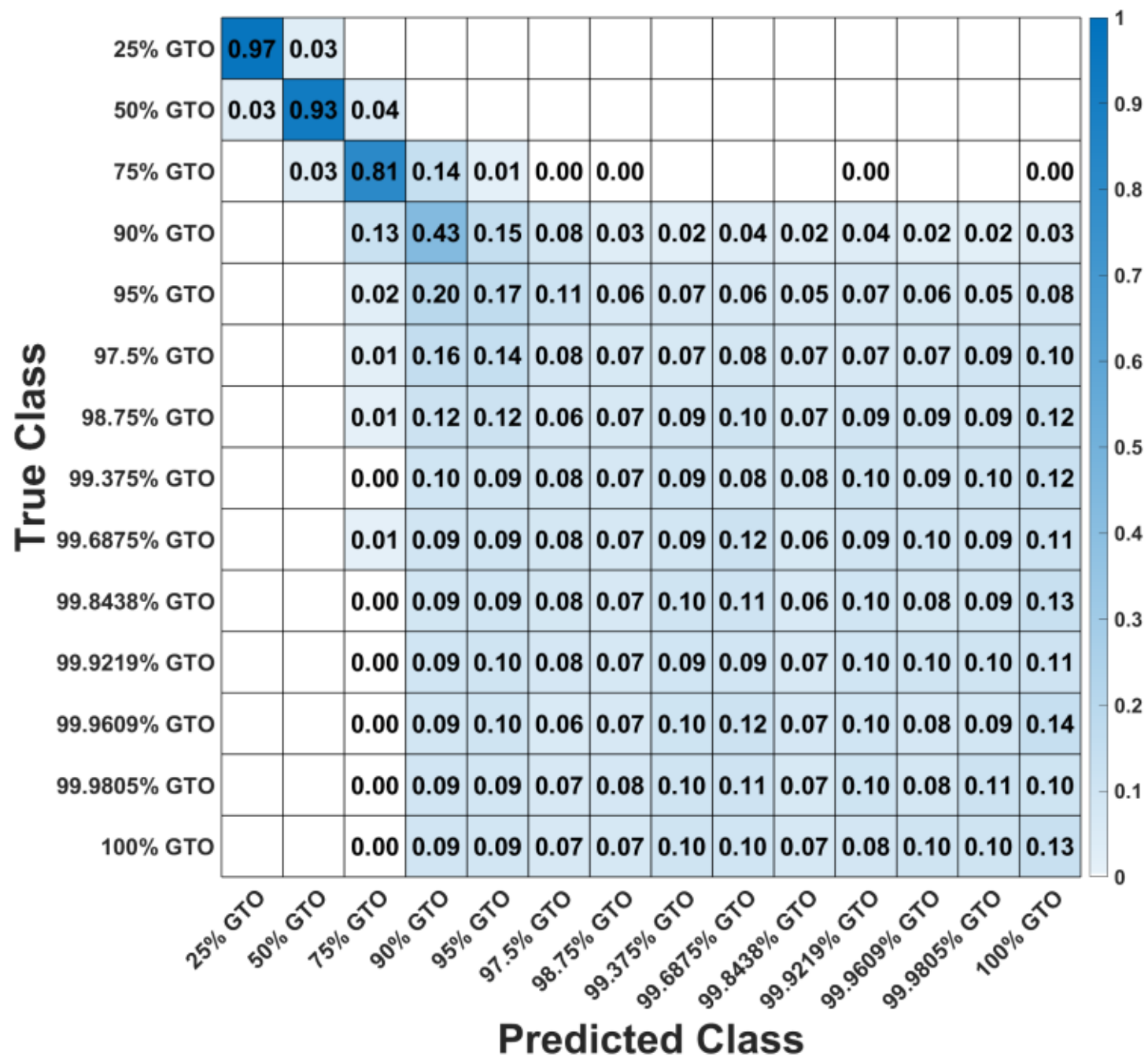

Figure S14 Row-wise normalized confusion matrix for multiclass classification of simulated mixtures, average standard deviation 9.14×10-3 (σ=60), using SVM-Linear.

Composition (Vol%)	AVERAGE STANDARD DEVIATION	
	Inter-day Samples	Intra-day Samples
**90% GTO 10 % OA**	4.71×10^{-4}	4.1×10^{-4}
**95% GTO 05 % OA**	4.64×10^{-4}	4.07×10^{-4}
**97.5% GTO 2.5 % OA**	4.82×10^{-4}	4.08×10^{-4}
**98.75% GTO 1.25 % OA**	4.8×10^{-4}	4.16×10^{-4}
**99.375% GTO 0.625 % OA**	4.77×10^{-4}	4.06×10^{-4}
**99.6875% GTO 0.3125 % OA**	4.73×10^{-4}	4.08×10^{-4}
**99.8438% GTO 0.1563 % OA**	4.72×10^{-4}	4.08×10^{-4}
**99.9219% GTO 0.0781 % OA**	4.7×10^{-4}	4.13×10^{-4}
**99.9609% GTO 0.0391 % OA**	4.66×10^{-4}	4.12×10^{-4}
**99.9805% GTO 0.0195 % OA**	4.61×10^{-4}	4.12×10^{-4}
**100% GTO**	4.49×10^{-4}	4.16×10^{-4}

Table S13 Average standard deviation value for inter-day and intra-samples for all the mixtures.

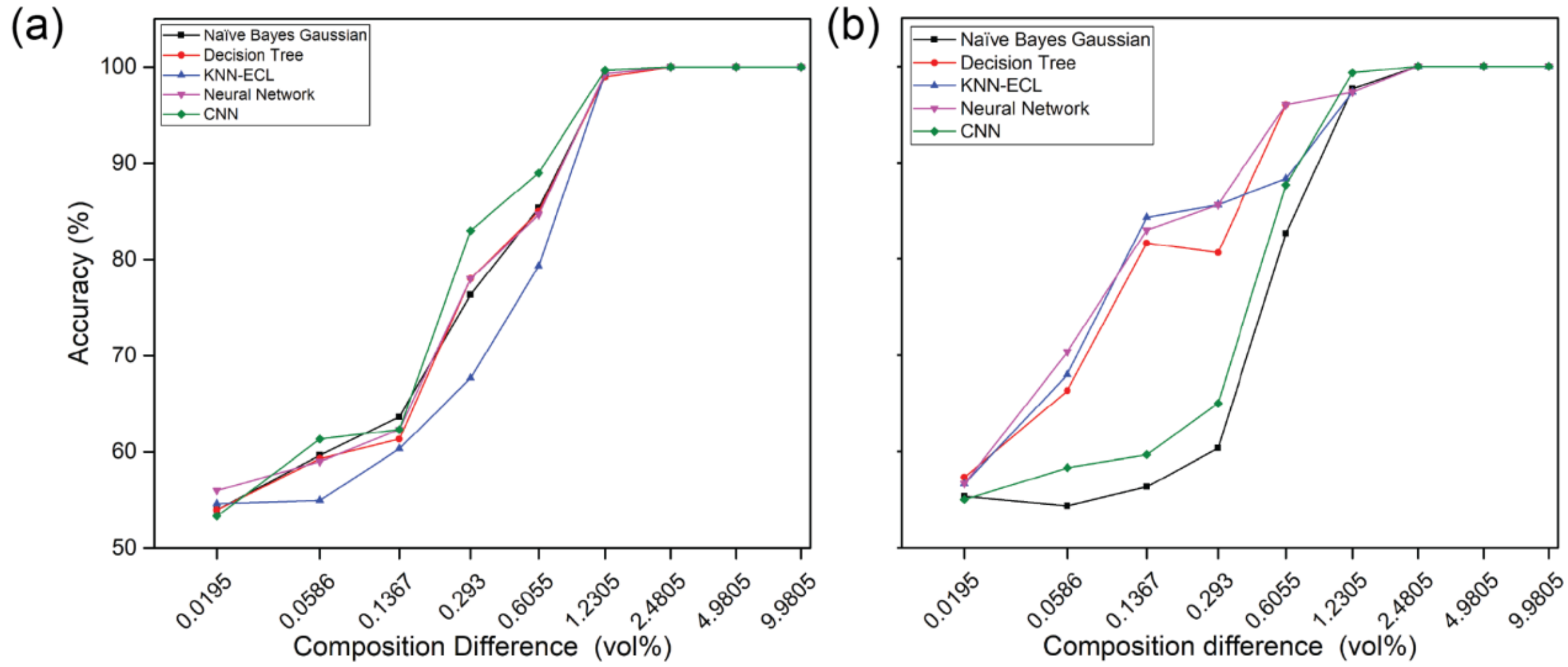

Figure S15 Classification accuracy for different composition differences (a) Results for intra-day measurements and (b) Results for inter-day measurements.

Composition difference (vol%)	Accuracy for Simulated Data	Accuracy for Experimental Data
**9.98046875**	100%	100%
**4.98046875**	100%	100%
**2.48046875**	100%	100%
**1.23046875**	100%	99.33%
**0.60546875**	96.1%	84.66%
**0.29296875**	74.4%	76.66%
**0.13671875**	56.25%	66%
**0.05859375**	52.5%	58.33%
**0.01953125**	52.7%	52.66%

Table S14 Comparison of classification accuracy using a Linear Support Vector Machine (SVM-Linear) for simulated versus experimental intra-day sample data.

Composition difference (vol%)	Accuracy for Simulated Data	Accuracy for Experimental Data
**9.98046875**	100%	100%
**4.98046875**	100%	100%
**2.48046875**	100%	100%
**1.23046875**	100%	97.66%
**0.60546875**	94.2%	80.66%
**0.29296875**	69.85%	63%
**0.13671875**	57.15%	59%
**0.05859375**	53.05%	54%
**0.01953125**	53.25%	57%

Table S15 Comparison of classification accuracy using a Linear Support Vector Machine (SVM-Linear) for simulated versus experimental inter-day sample data.

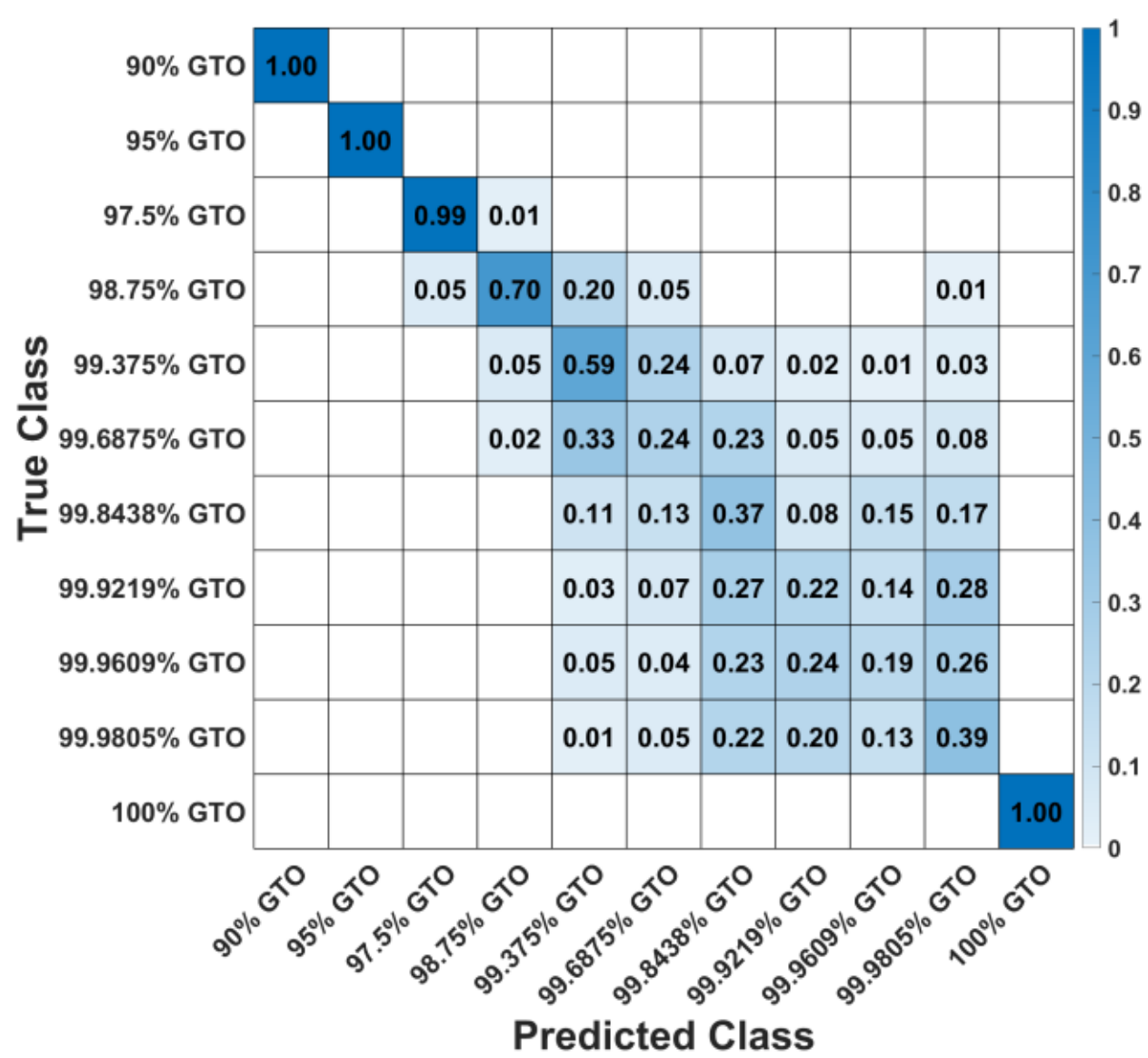

Figure S16 Row-wise normalized confusion matrix for multiclass classification of intra-day data using Naïve Bayes Gaussian.

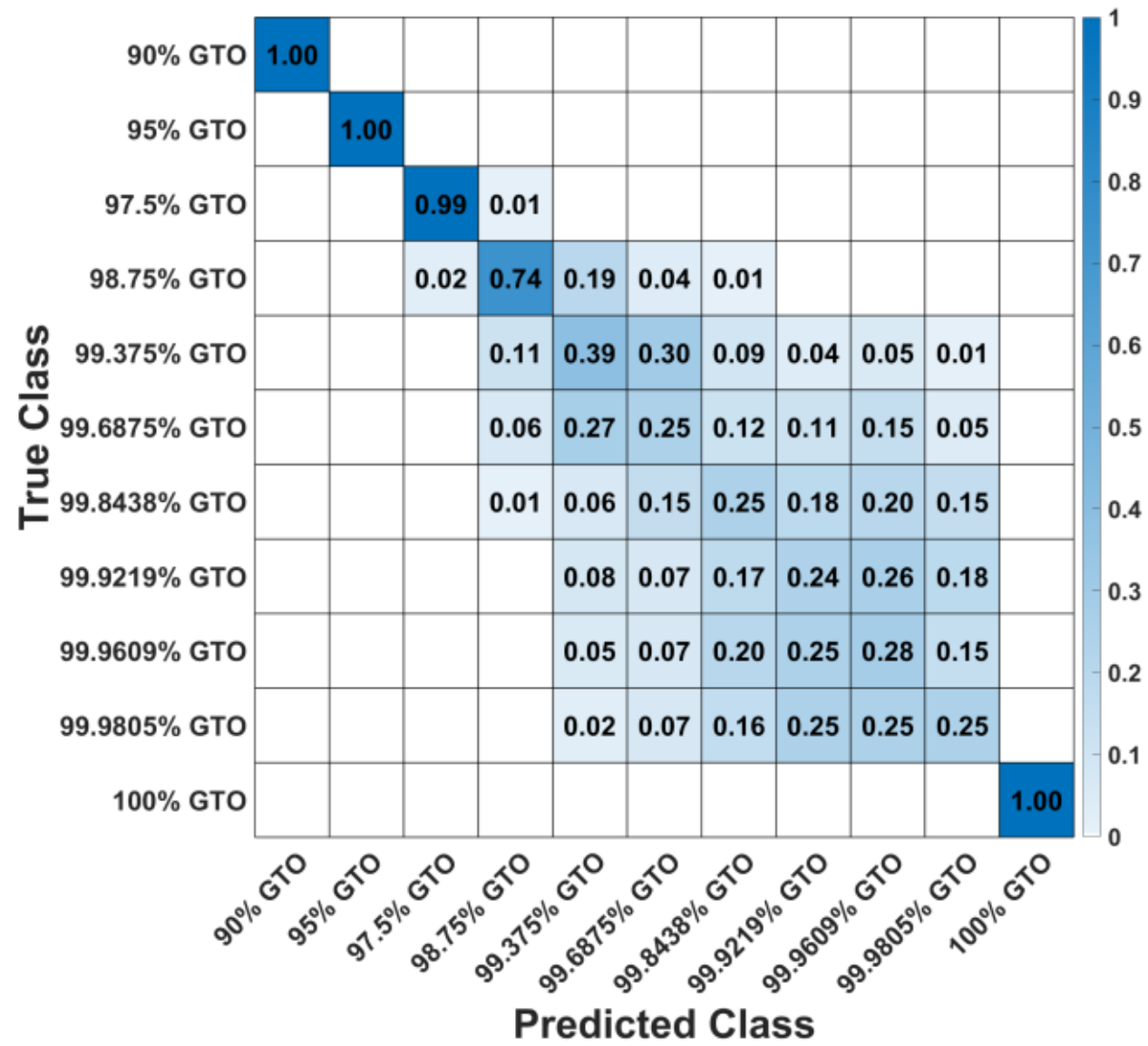

Figure S17 Row-wise normalized confusion matrix for multiclass classification of intra-day data using Decision Tree.

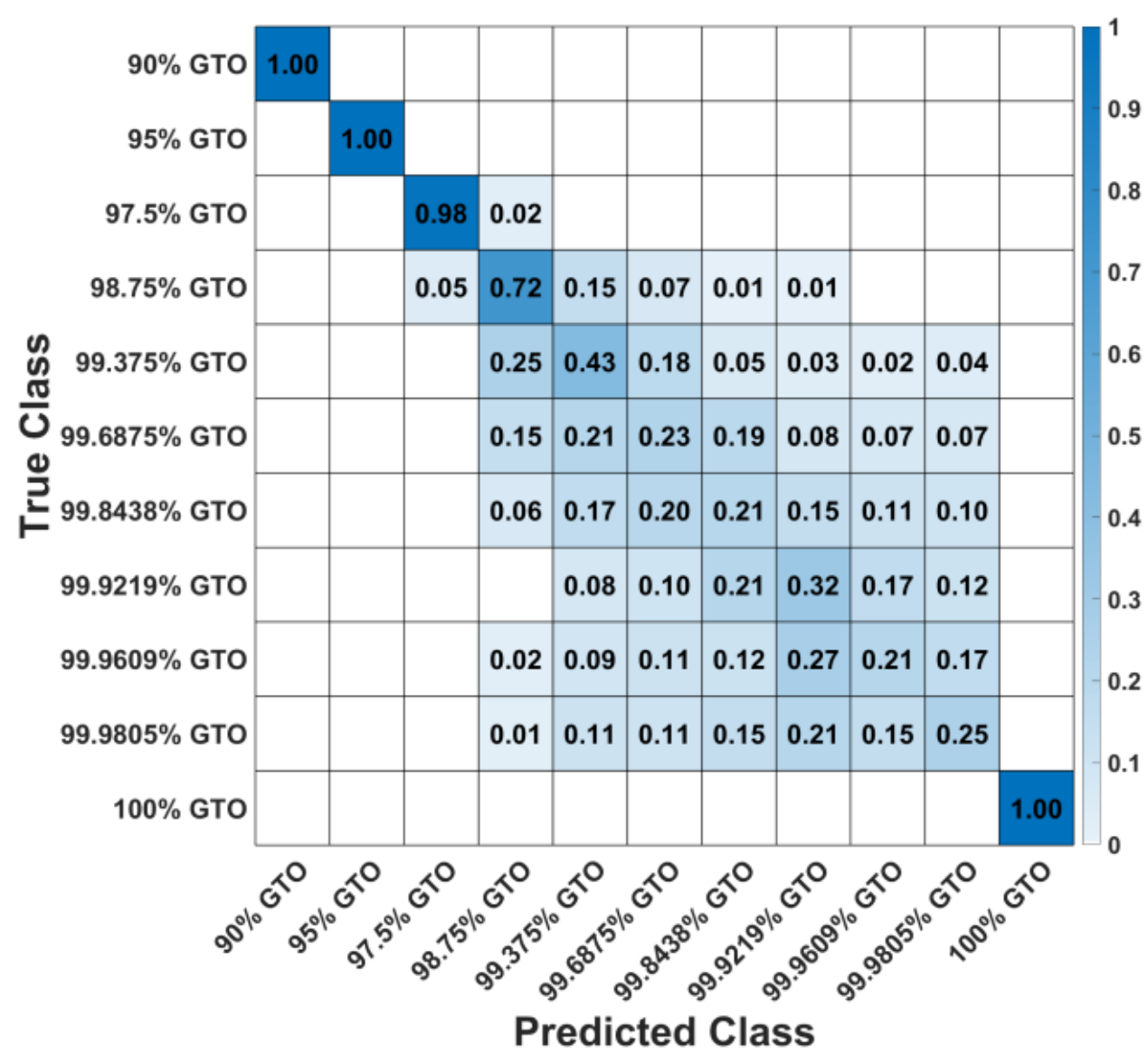

Figure S18 Row-wise normalized confusion matrix for multiclass classification of intra-day data using KNN-Euclidean.

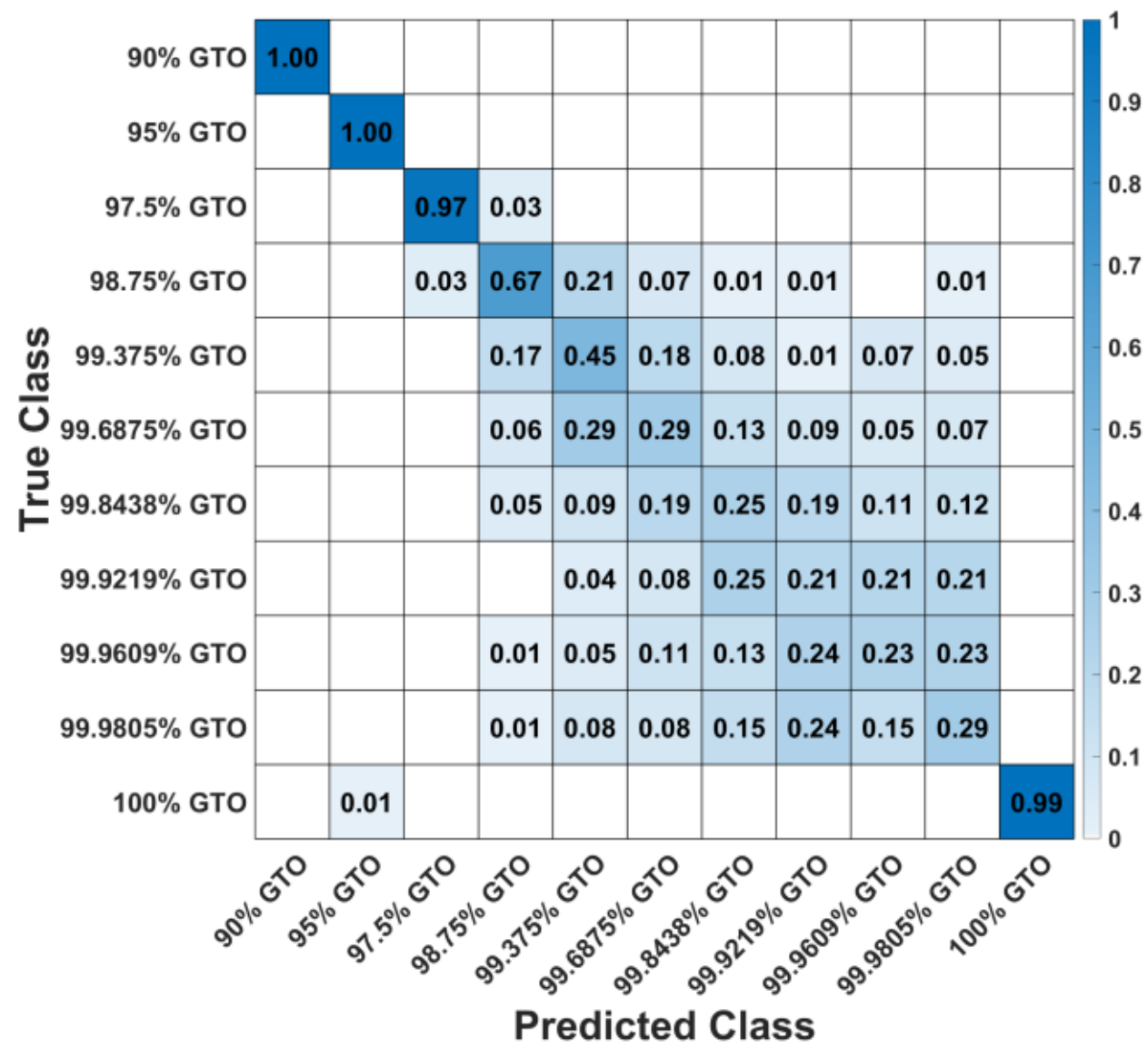

Figure S19 Row-wise normalized confusion matrix for multiclass classification of intra-day data using Neural Network.

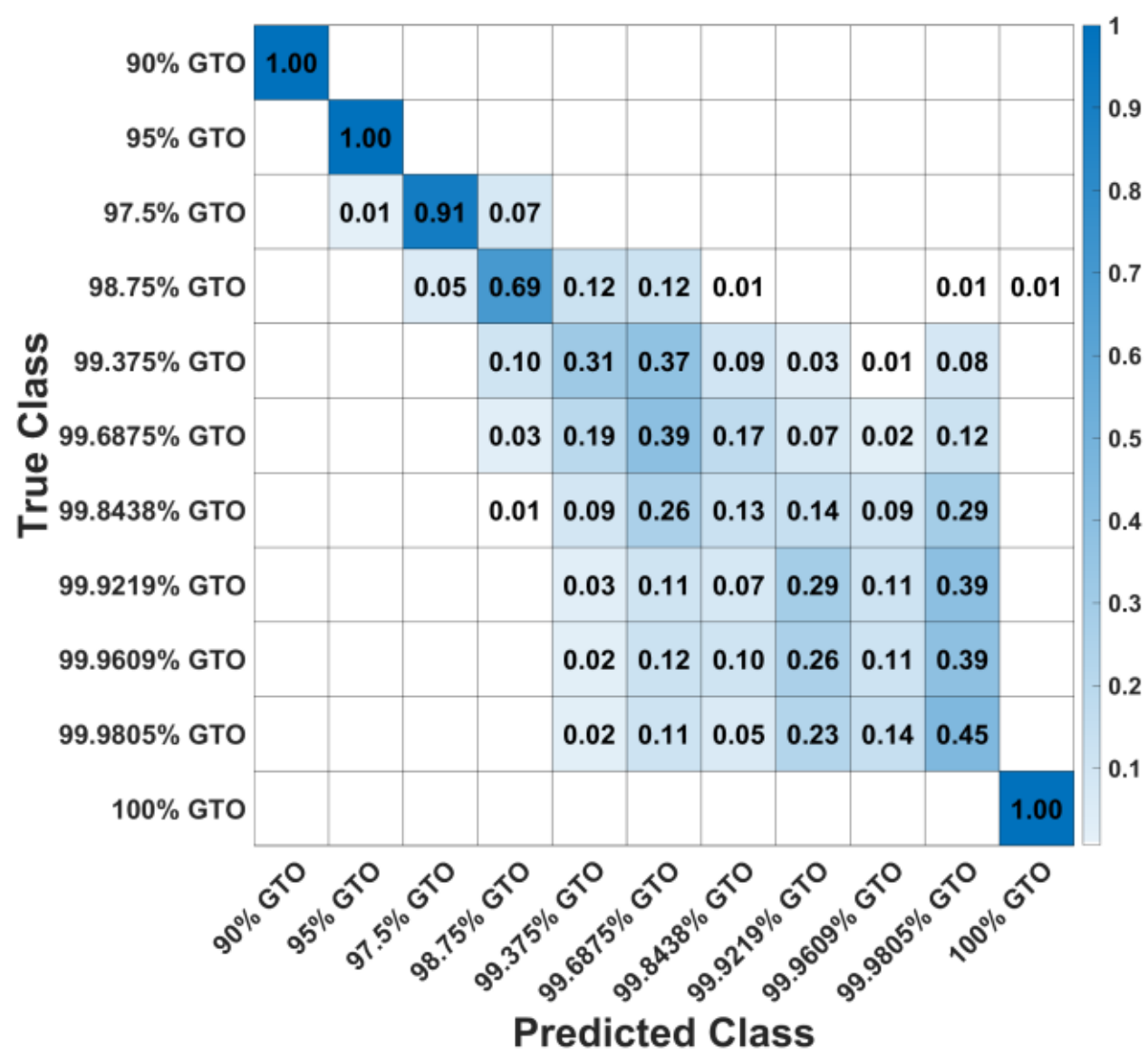

Figure S20 Row-wise normalized confusion matrix for multiclass classification of intra-day data using Convolutional Neural Network.

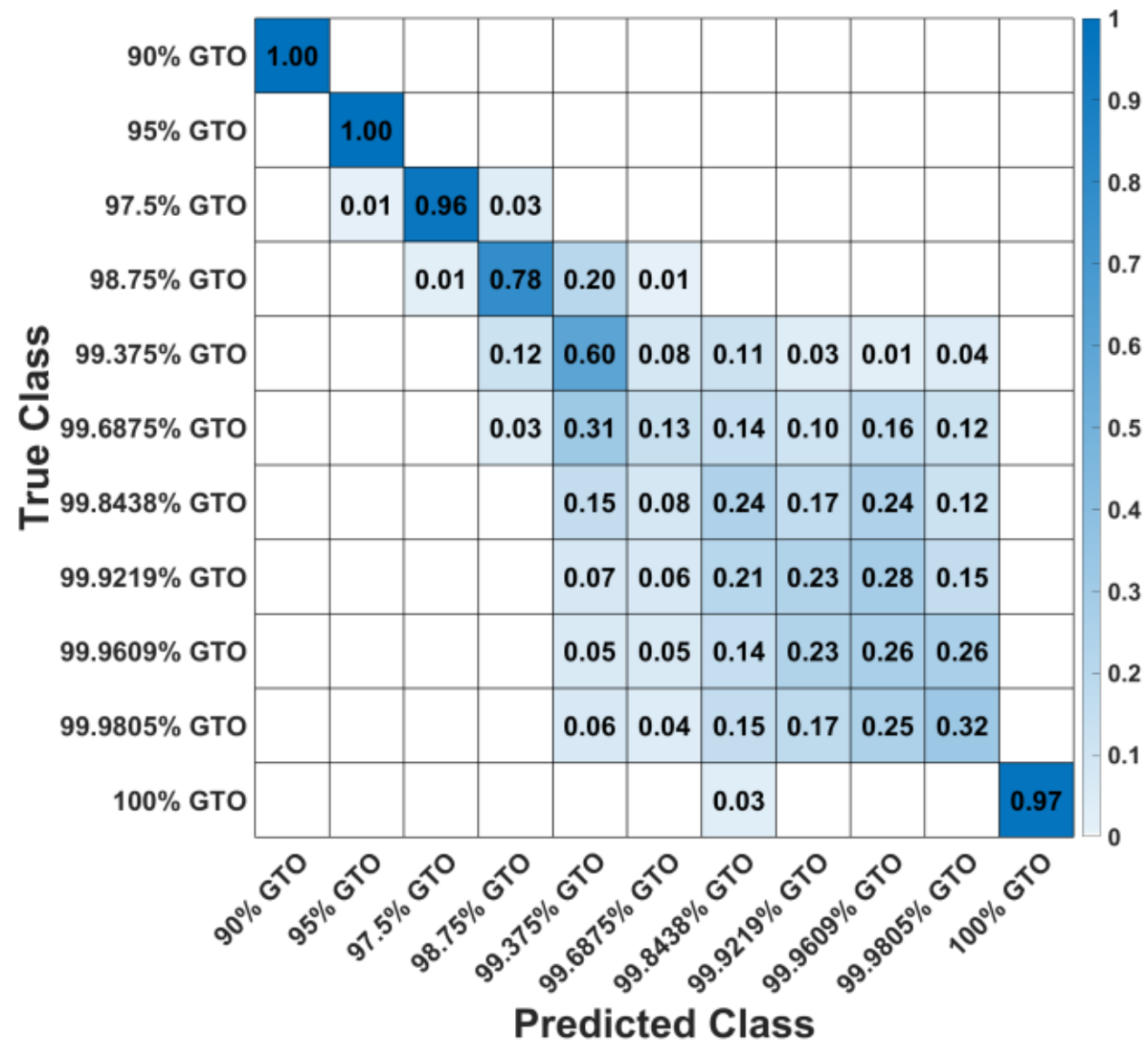

Figure S21 Row-wise normalized confusion matrix for multiclass classification of inter-day data using Naïve Bayes Gaussian.

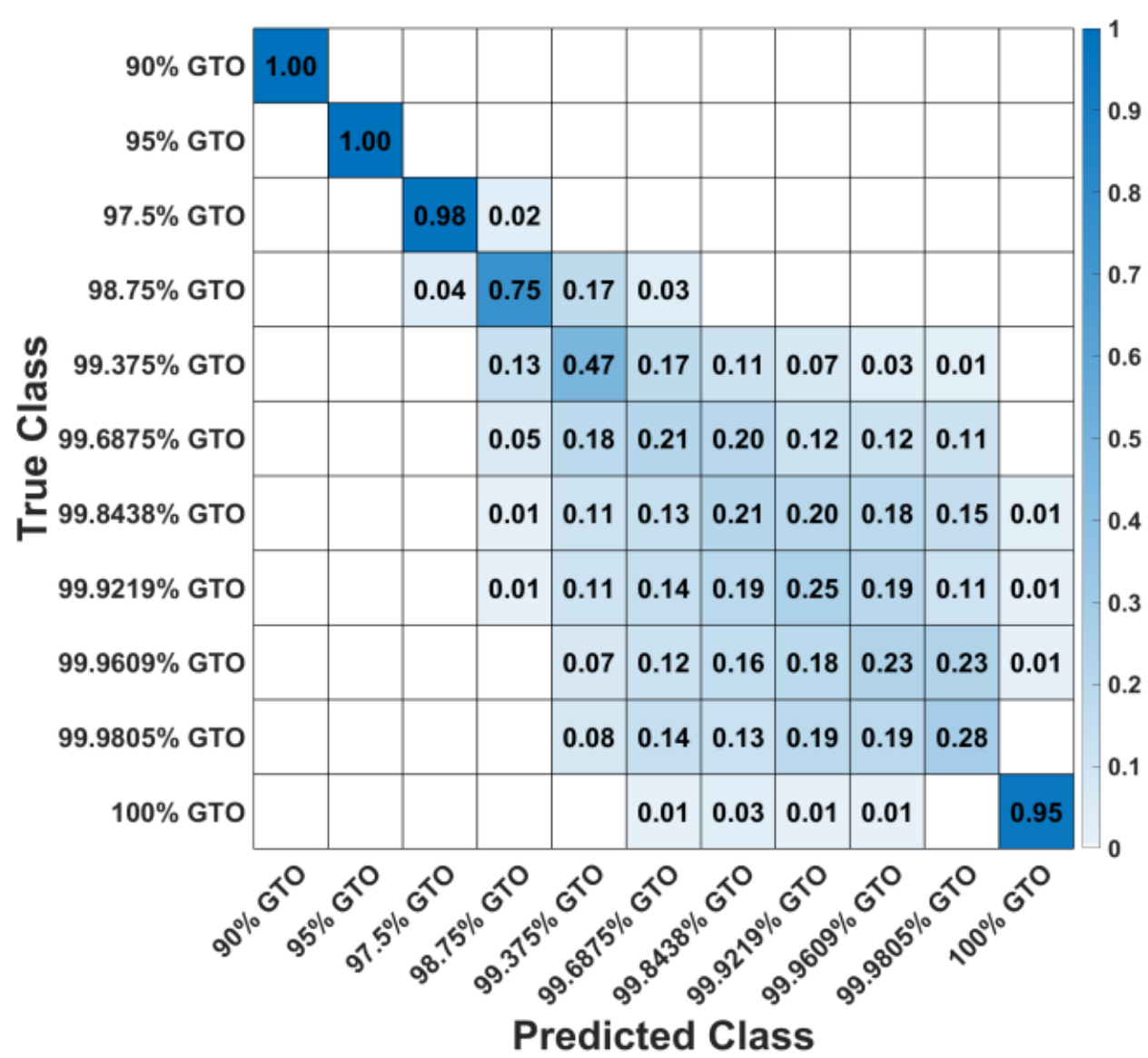

Figure S22 Row-wise normalized confusion matrix for multiclass classification of inter-day data using Decision Tree.

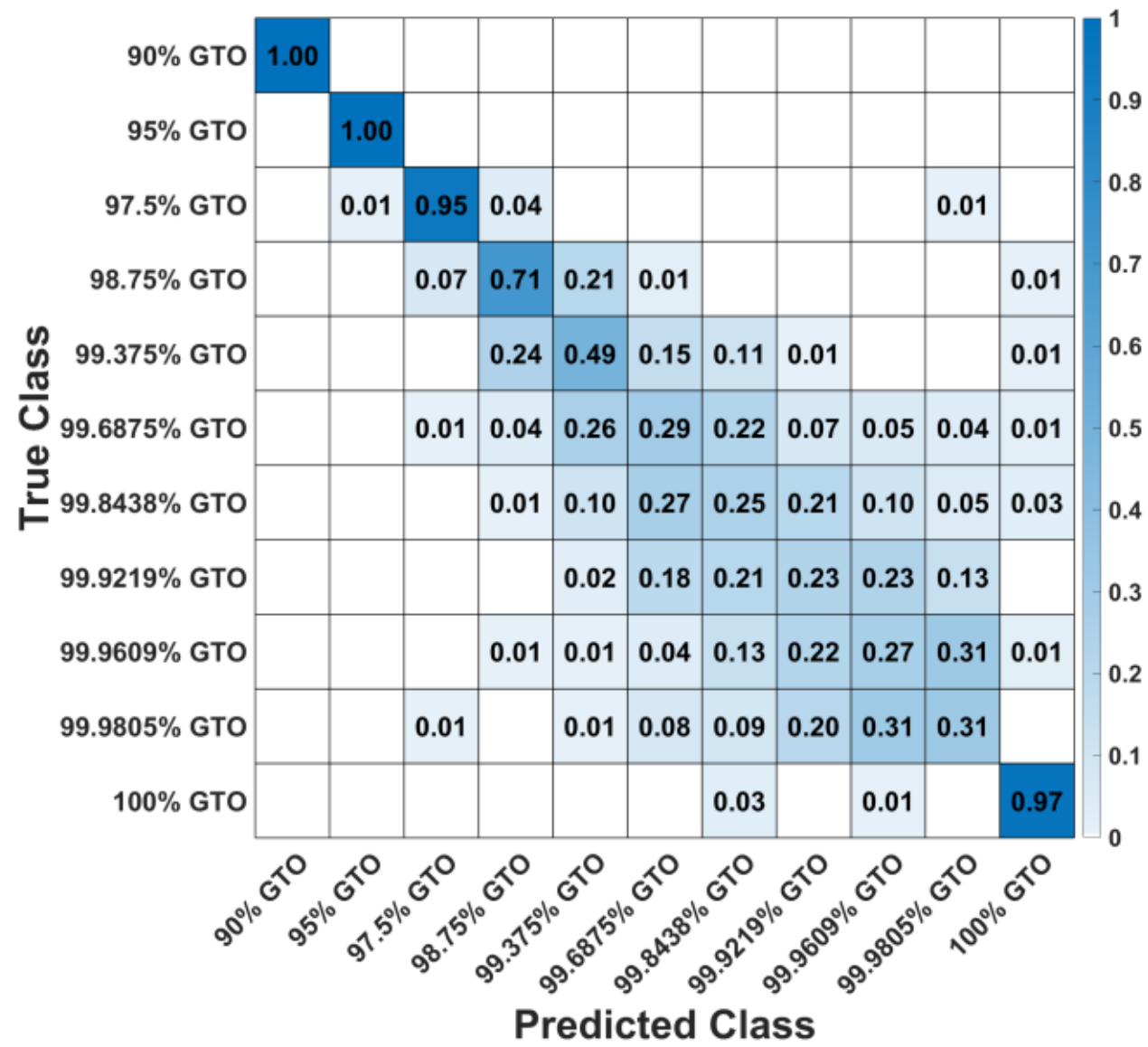

Figure S23 Row-wise normalized confusion matrix for multiclass classification of inter-day data using KNN-Euclidean.

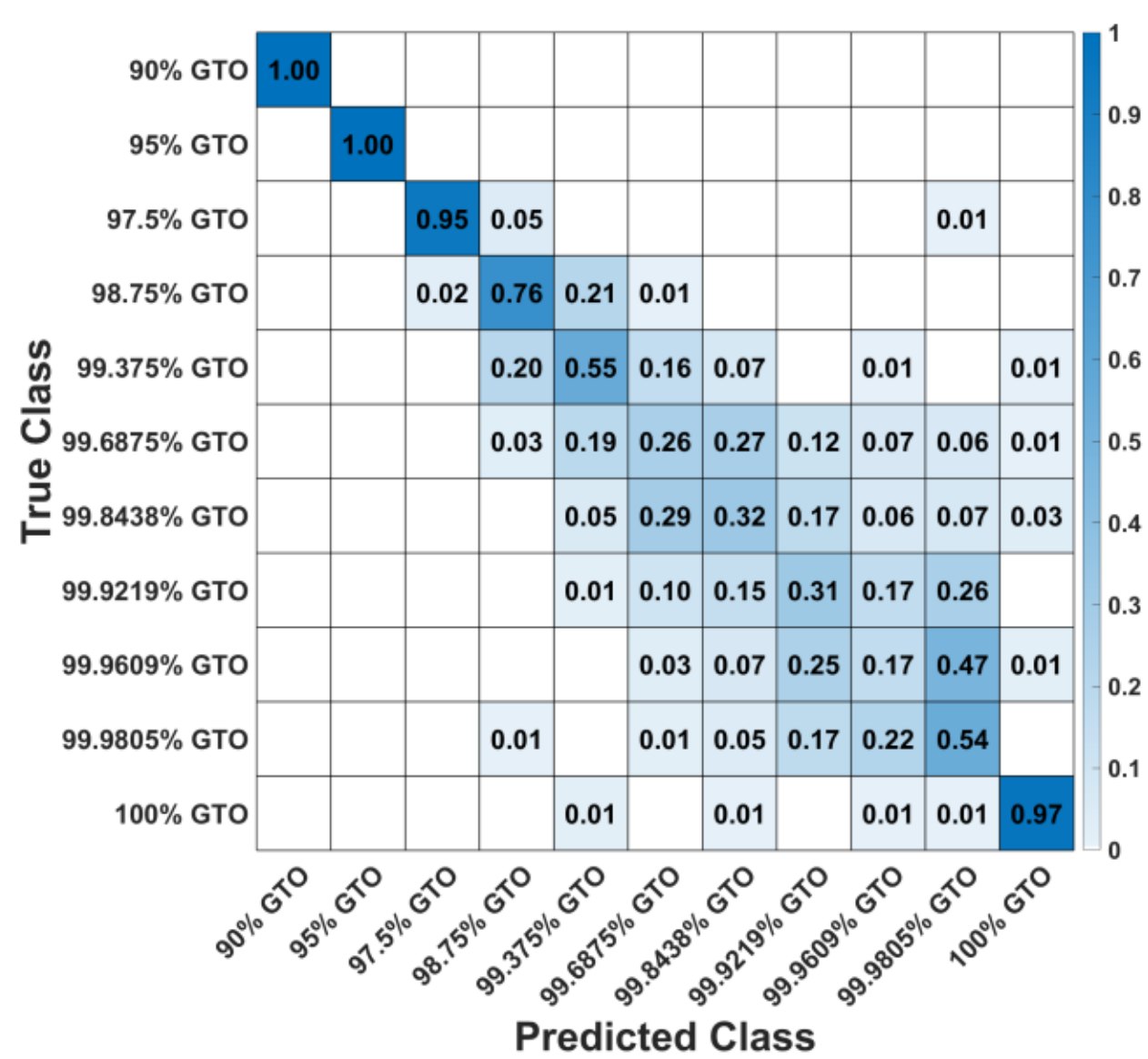

Figure S24 Row-wise normalized confusion matrix for multiclass classification of inter-day data using Neural Network.

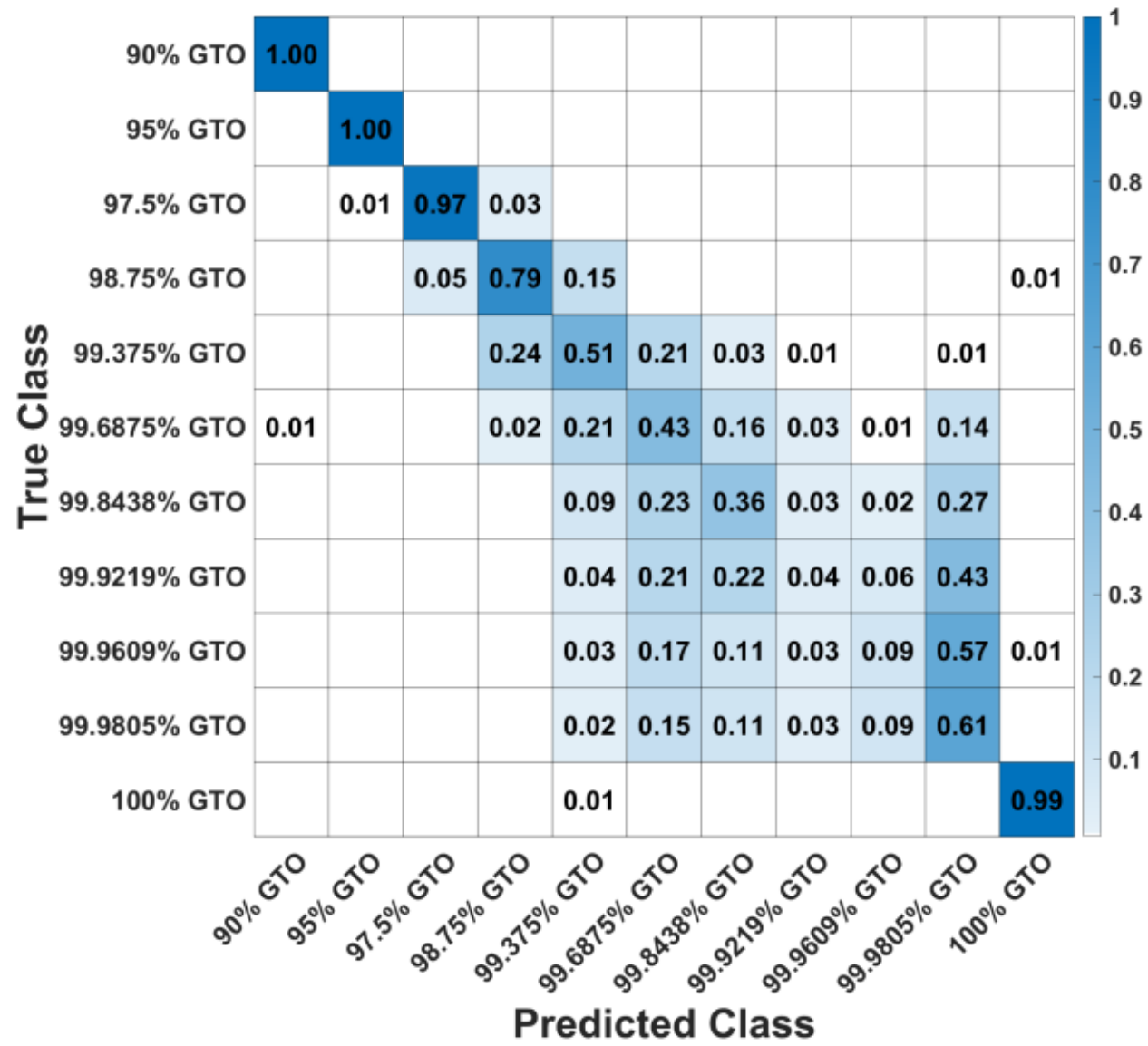

Figure S25 Row-wise normalized confusion matrix for multiclass classification of inter-day data using Convolutional Neural Network.

LABELS	GENOTYPE INFORMATION	SOURCE
E. coli	*E. coli* BL21(DE3) F^- *dcm ompT hsdS*(r_B^- m_B^-) *gal* λ(DE3)	This Paper
EBY100	MATa *AGA1*::*GAL1-AGA1*::*URA3 ura3-52 trp1 le*u2Δ*1 his*3Δ*200 pep4*::*HIS3 prb1*Δ*1.6R can1* GAL	Tsai et al. 2009[5]
L. lactis	MG1363	This Paper
L. reuteri	ATCC BAA-2837 (IDCC 3701)	ATCC
YLH2	GSY1136 YIplac211YB/I/E* Δctt1	Godara et al. 2019 [6]
YAG01	GSY1136 YIplac211YB/I/E* Δctt1, HIS7 389	Godara et al. 2019 [6]
YAG02	GSY1136 YIplac211YB/I/E* Δctt1, SRO9/GFD2 int	Godara et al. 2019 [6]
YAG03	GSY1136 YIplac211YB/I/E* Δctt1, TYE7 86	Godara et al. 2019 [6]
YAG04	GSY1136 YIplac211YB/I/E* Δctt1, FLO1 925	Godara et al. 2019 [6]
YAG05	GSY1136 YIplac211YB/I/E* Δctt1, DAK2/AQY3 int	Godara et al. 2019 [6]
YAG06	GSY1136 YIplac211YB/I/E* Δctt1, SCY1 1836	Godara et al. 2019 [6]
YAG07	GSY1136 YIplac211YB/I/E* Δctt1, EPL1 1754	Godara et al. 2019 [6]
YAG08	GSY1136 YIplac211YB/I/E* Δctt1, ALG6 1411	Godara et al. 2019 [6]
YAG09	GSY1136 YIplac211YB/I/E* Δctt1, MDS3 ins	Godara et al. 2019 [6]
YAG10	GSY1136 YIplac211YB/I/E* Δctt1, YMRCTy1-3 1078	Godara et al. 2019 [6]
YAG17	GSY1136 YIplac211YB/I/E* Δctt1, ALG6 1411, EPL1 1754	Godara et al. 2019 [6]
YAG20	GSY1136 YIplac211YB/I/E* Δctt1, EPL1 1754, DAK2/AQY3 int	Godara et al. 2019 [6]
YAG22	GSY1136 YIplac211YB/I/E* Δctt1, ALG6 1411, DAK2/AQY3 int	Godara et al. 2019 [6]
YAG23	GSY1136 YIplac211YB/I/E* Δctt1, ALG6 1411, DAK2/AQY3 int, EPL1 1754	Godara et al. 2019 [6]
YAG28	GSY1136 YIplac211YB/I/E* Δctt1, ALG6 1411, DAK2/AQY3 int, YMRCTy1-3 1078	Godara et al. 2019 [6]

Table S16 Strain and mutation label details of microorganisms used in the study.

**Label**	**Average standard deviation**
E. coli	1.01×10^{-2}
EBY100	8.38×10^{-3}
L. lactis	1.11×10^{-2}
L. reuteri	9.37×10^{-3}
YAG01	5.98×10^{-3}
YAG02	4.05×10^{-3}
YAG03	4.32×10^{-3}
YAG04	4.9×10^{-3}
YAG05	4.5×10^{-3}
YAG06	7.02×10^{-3}
YAG07	4.67×10^{-3}
YAG08	5.78×10^{-3}
YAG09	5.25×10^{-3}
YAG10	5.8×10^{-3}
YAG17	4.04×10^{-3}
YAG20	4.48×10^{-3}
YAG22	3.46×10^{-3}
YAG23	4.08×10^{-3}
YAG28	6.84×10^{-3}
YLH2	3.65×10^{-3}

Table S17 Average standard deviation of Raman spectra in each microorganism.

Label	PC	PD
**E. coli**	-0.09431	0.207603
**EBY100**	-0.073745	0.19612
**L. lactis**	-0.030124	0.202841
**L. reuteri**	-0.125977	0.207085
**YAG01**	0.921848	0.037648
**YAG02**	0.95351	0.031026
**YAG03**	0.950231	0.032904
**YAG04**	0.941429	0.034914
**YAG05**	0.952713	0.032311
**YAG06**	0.900465	0.041817
**YAG07**	0.949683	0.031798
**YAG08**	0.93822	0.034172
**YAG09**	0.940327	0.032547
**YAG10**	0.928058	0.040699
**YAG17**	0.953059	0.031008
**YAG20**	0.934264	0.05119
**YAG22**	0.959237	0.0342
**YAG23**	0.955394	0.029217
**YAG28**	0.814117	0.103241

Table S18 Similarity scores for spectra of various mutated yeast strains and other microorganisms compared to the industrial strain YLH2. Green indicates high similarity, while red represents dissimilarity.

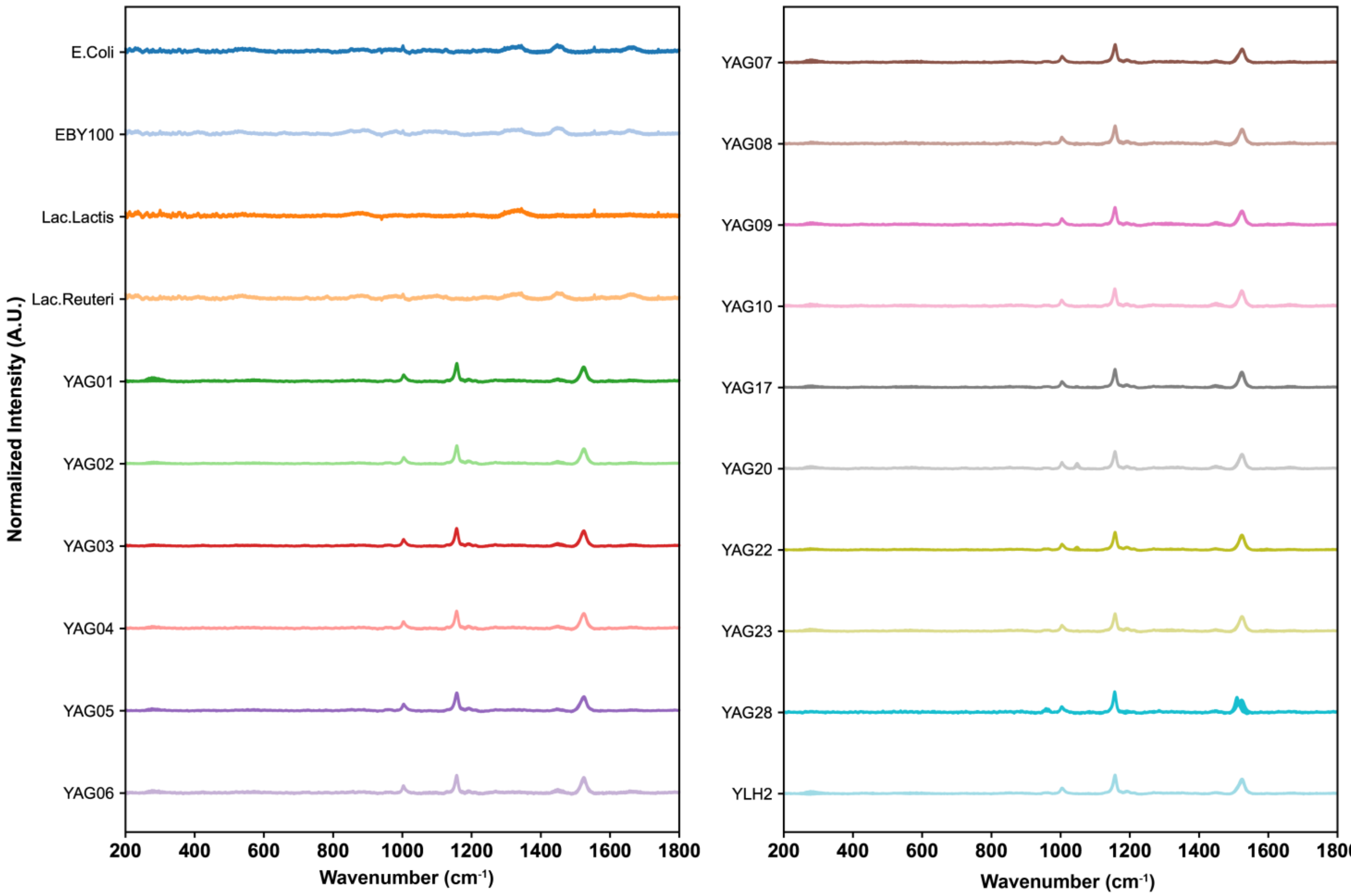

Figure S26 Mean spectra and double standard deviations (shaded region) for E. coli, EBY100, L. lactis, L. reuteri, YAG01, YAG02, YAG03, YAG04, YAG05, YAG06, YAG07, YAG08, YAG09, YAG10, YAG17, YAG20, YAG22, YAG23, YAG28, and YLH2.

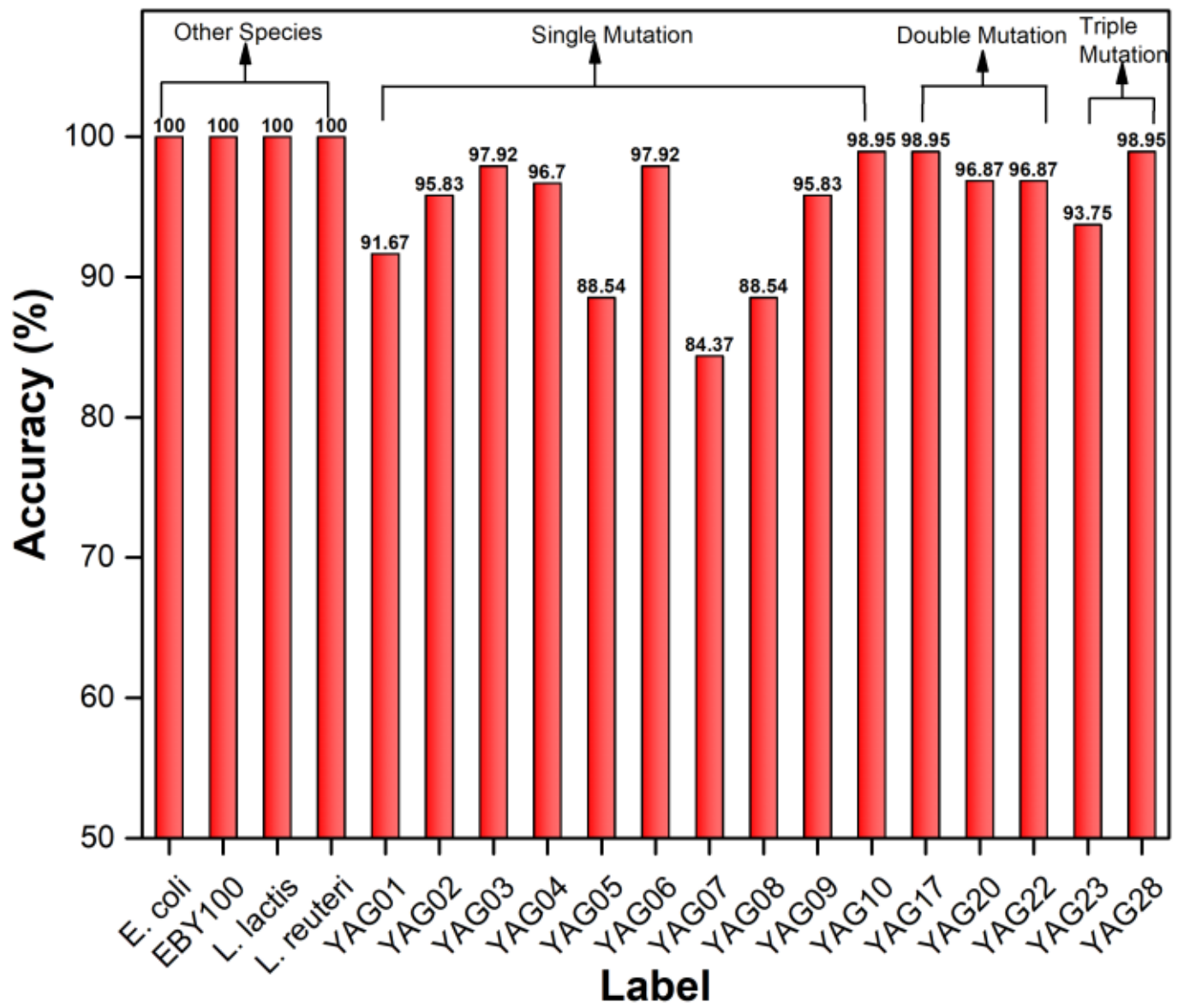

Figure S27 Binary classification results for all the cells compared to YLH2 using Naïve Bayes Gaussian.

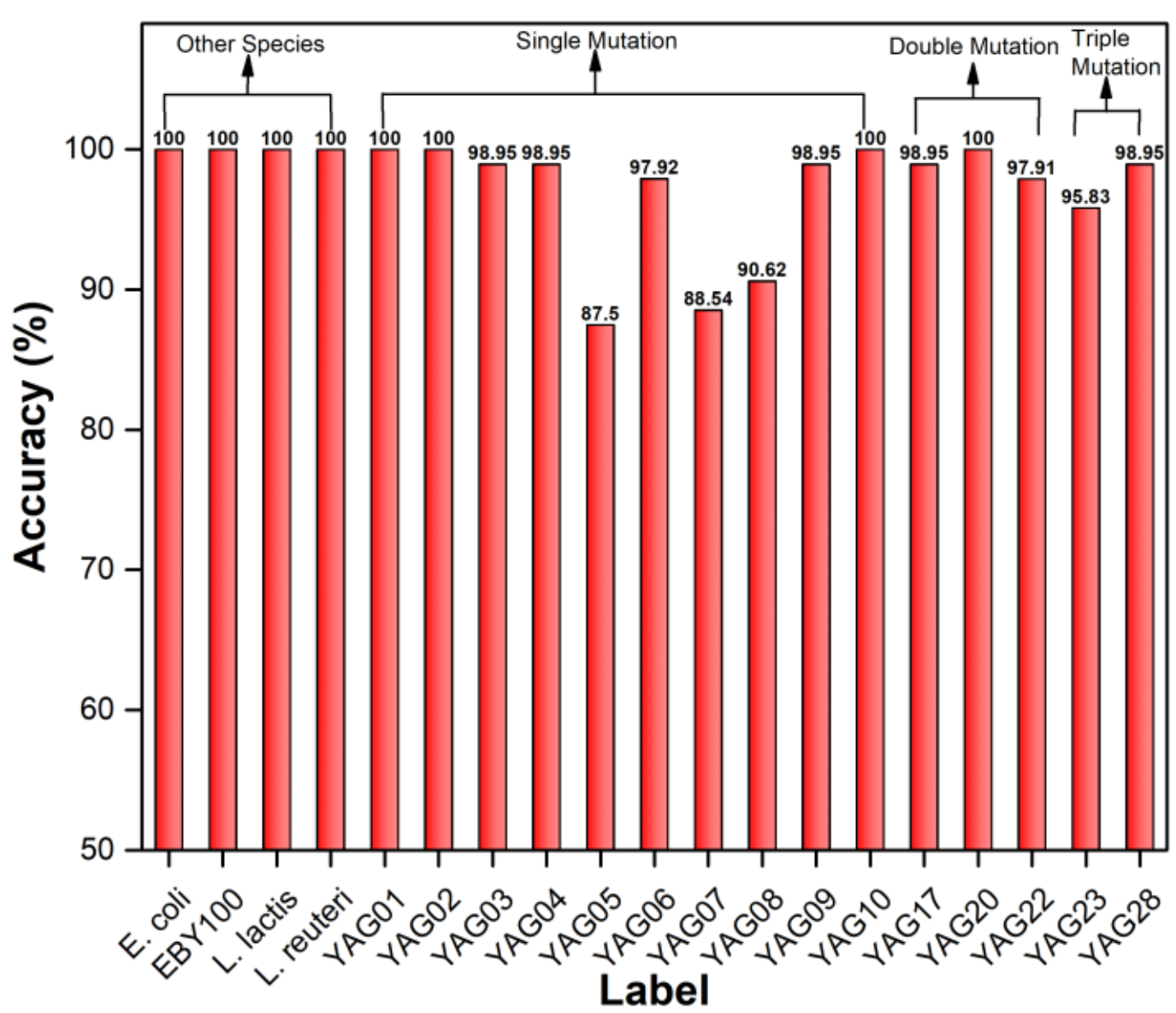

Figure S28 Binary classification results for all the cells compared to YLH2 using Decision Tree.

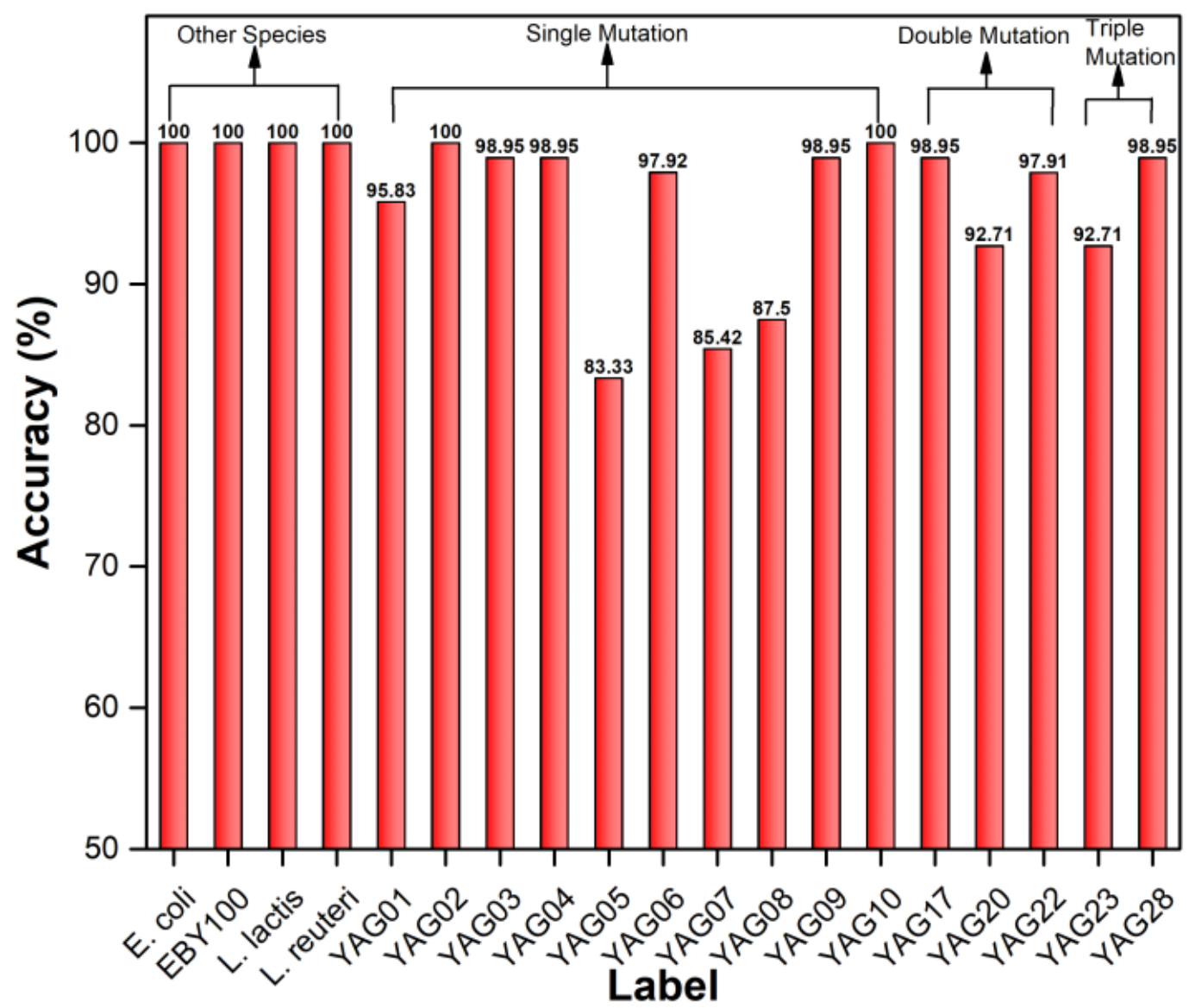

Figure S29 Binary classification results for all the cells compared to YLH2 using KNN-Euclidean.

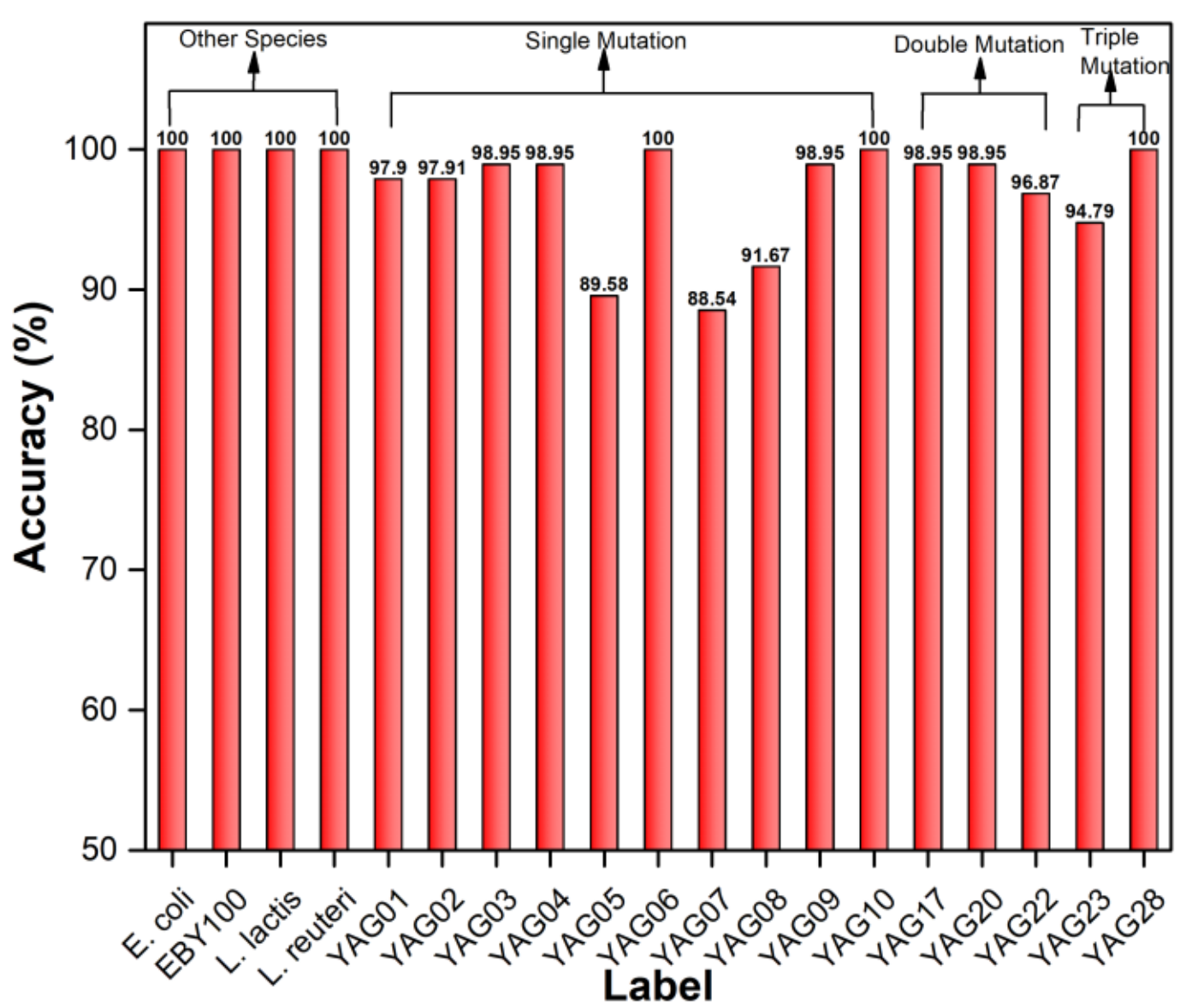

Figure S30 Binary classification results for all the cells compared to YLH2 using Neural Network.

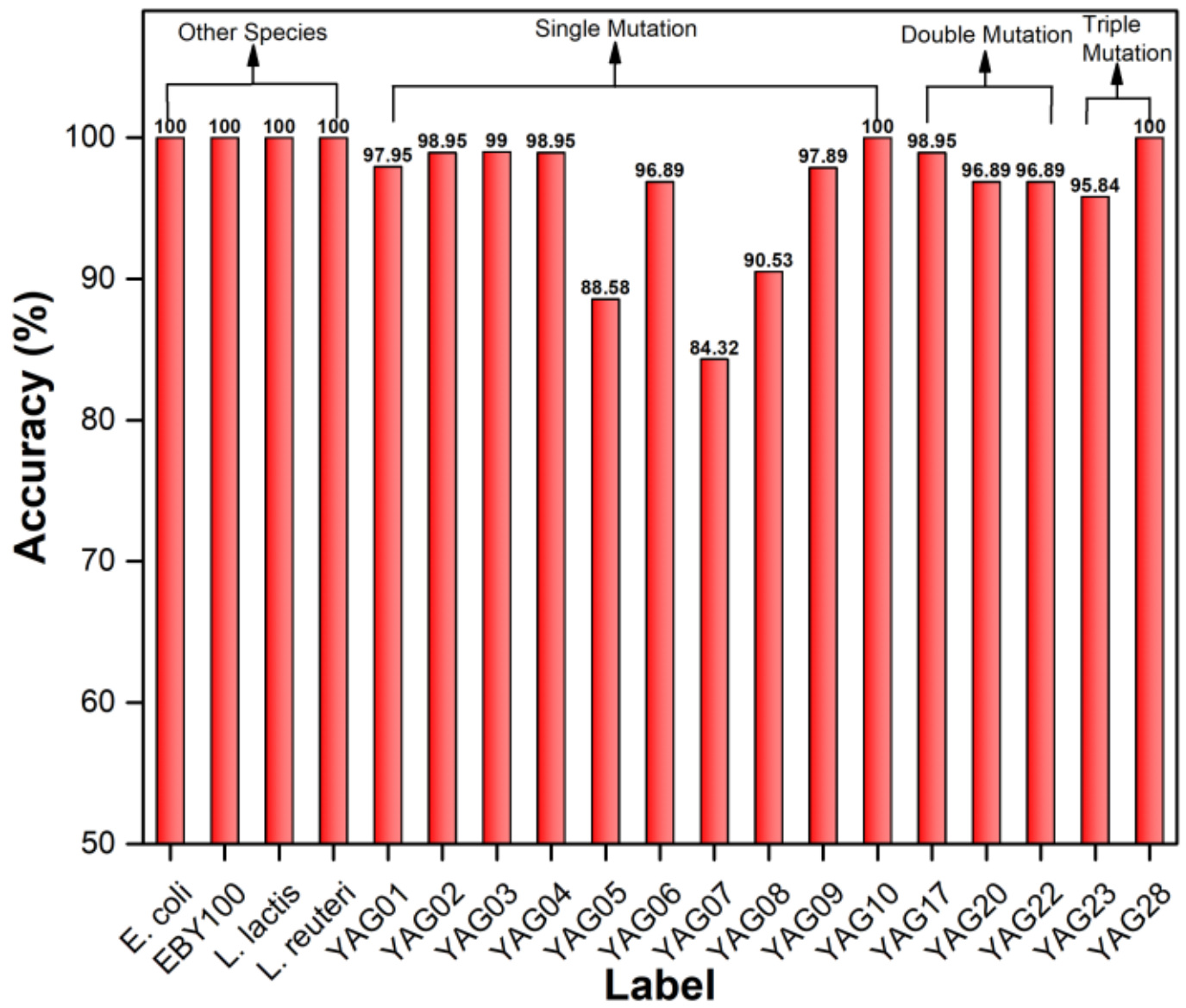

Figure S31 Binary classification results for all the cells compared to YLH2 using Convolutional Neural Network.

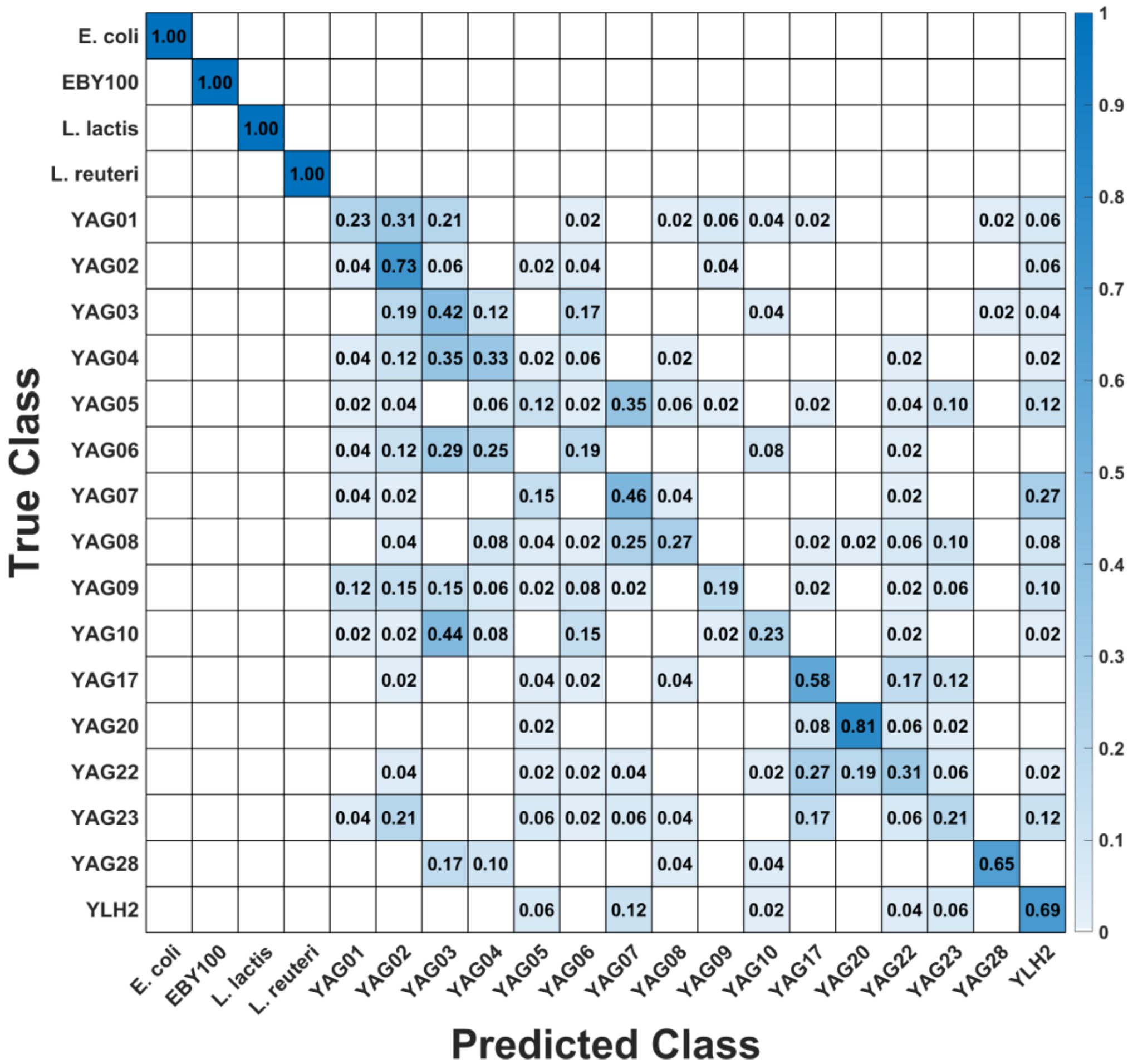

Figure S32 Row-wise normalized confusion matrix for multiclass classification of cells using Naïve Bayes Gaussian (Overall accuracy: 52.08%).

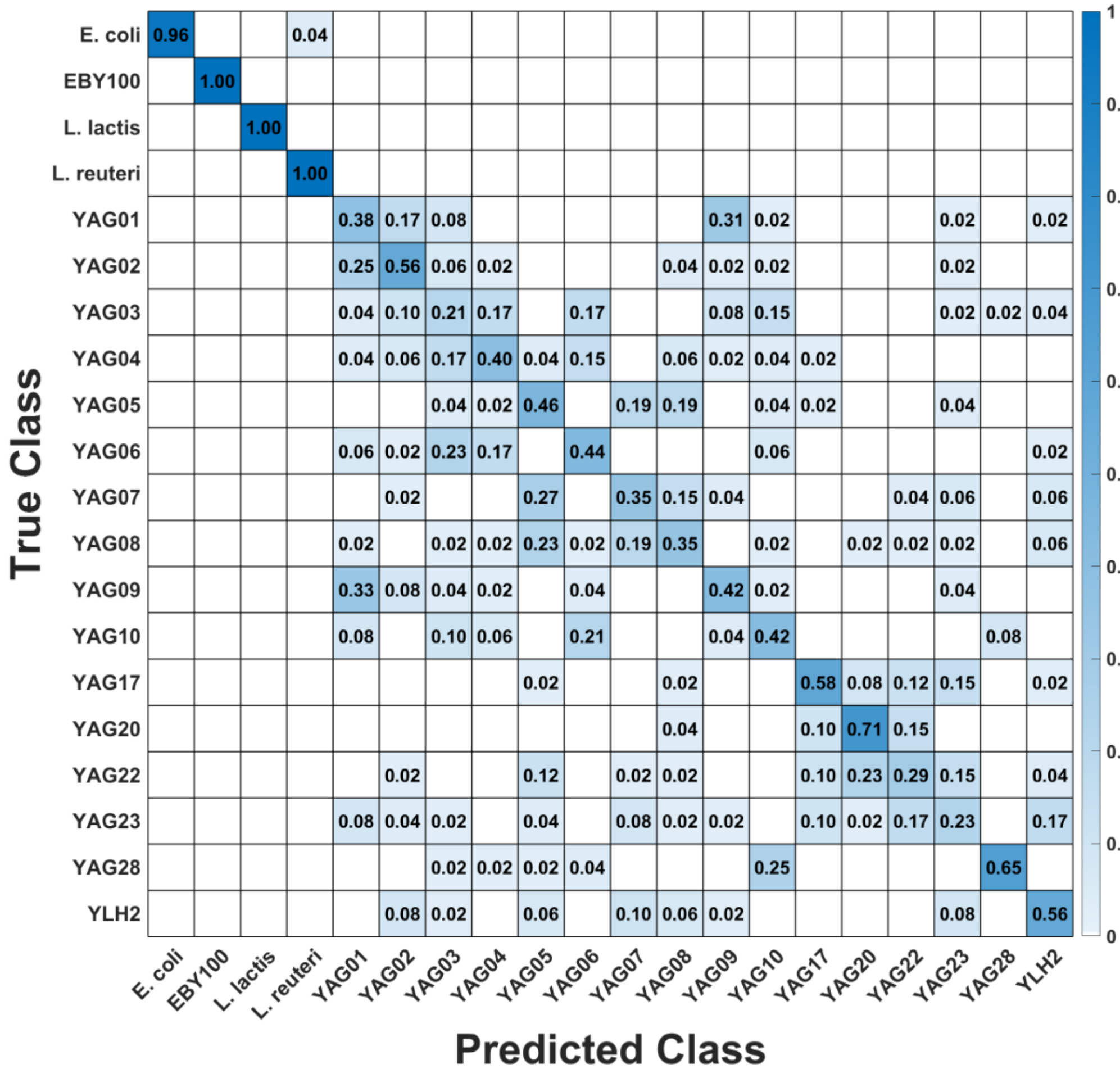

Figure S33 Row-wise normalized confusion matrix for multiclass classification of cells using a Decision Tree (Overall accuracy: 54.79%).

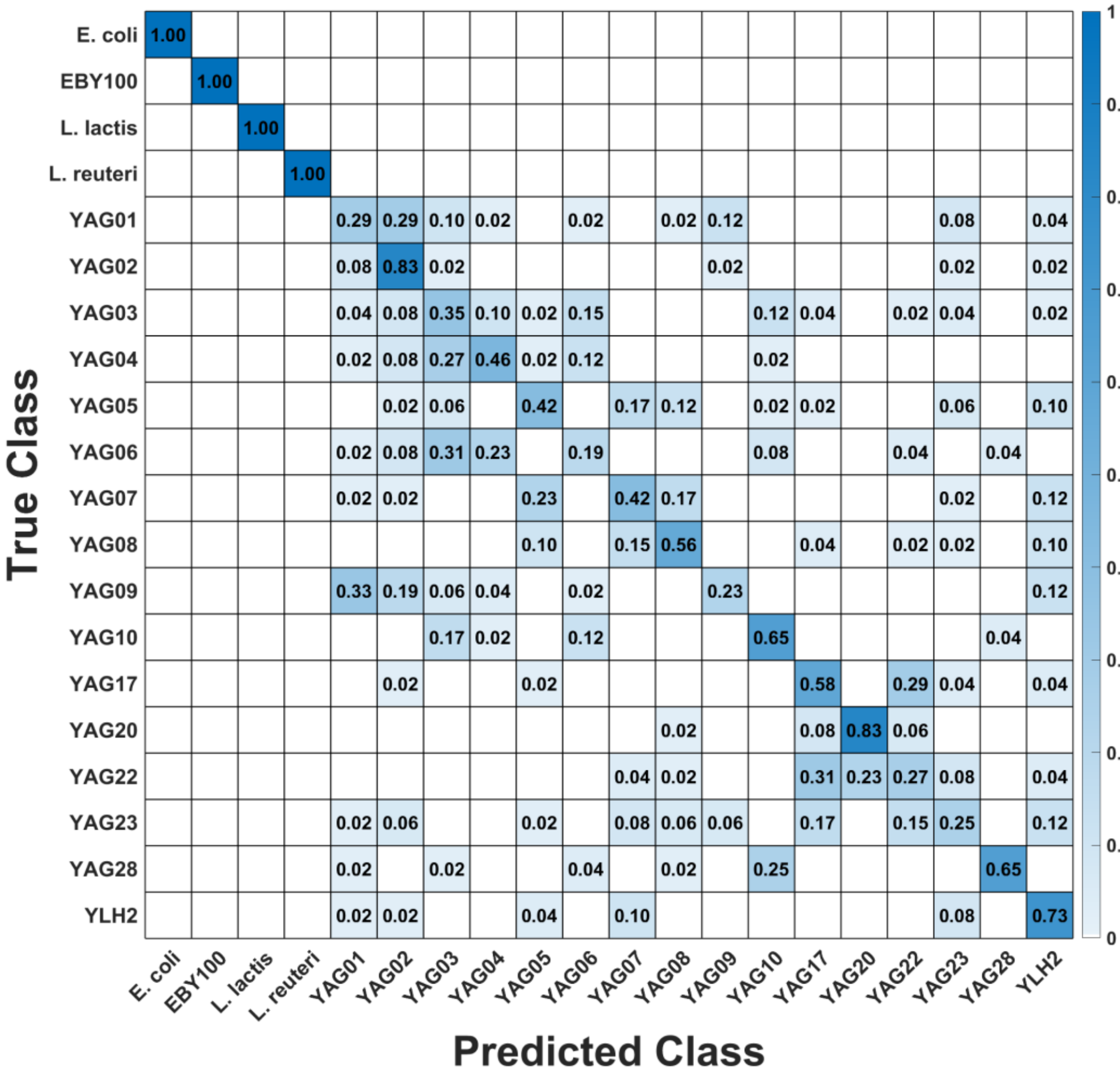

Figure S34 Row-wise normalized confusion matrix for multiclass classification of cells using KNN-Euclidean (Overall accuracy: 55.76%).

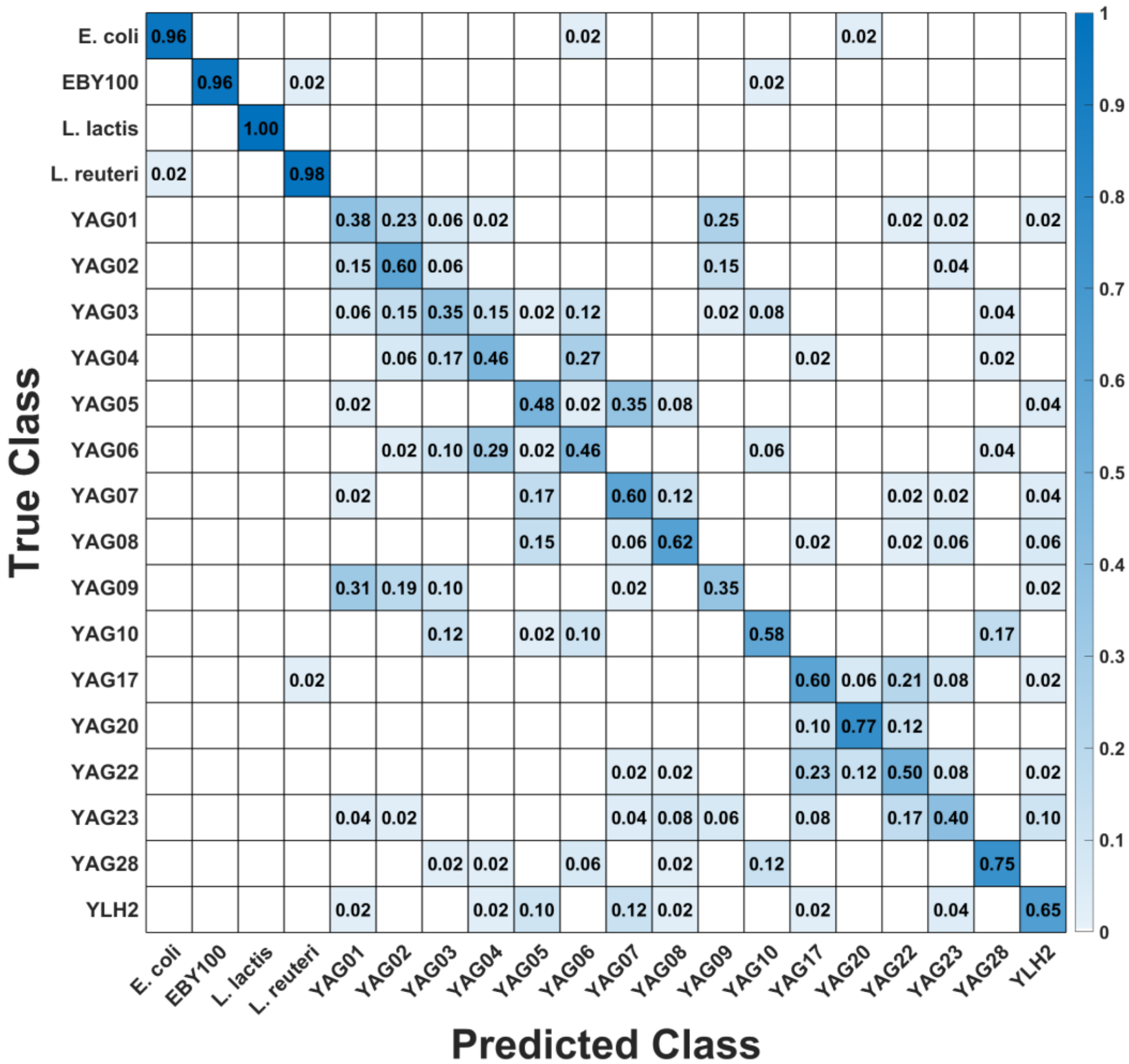

Figure S35 Row-wise normalized confusion matrix for multiclass classification of cells using a Neural network (Overall accuracy- 62.29%).

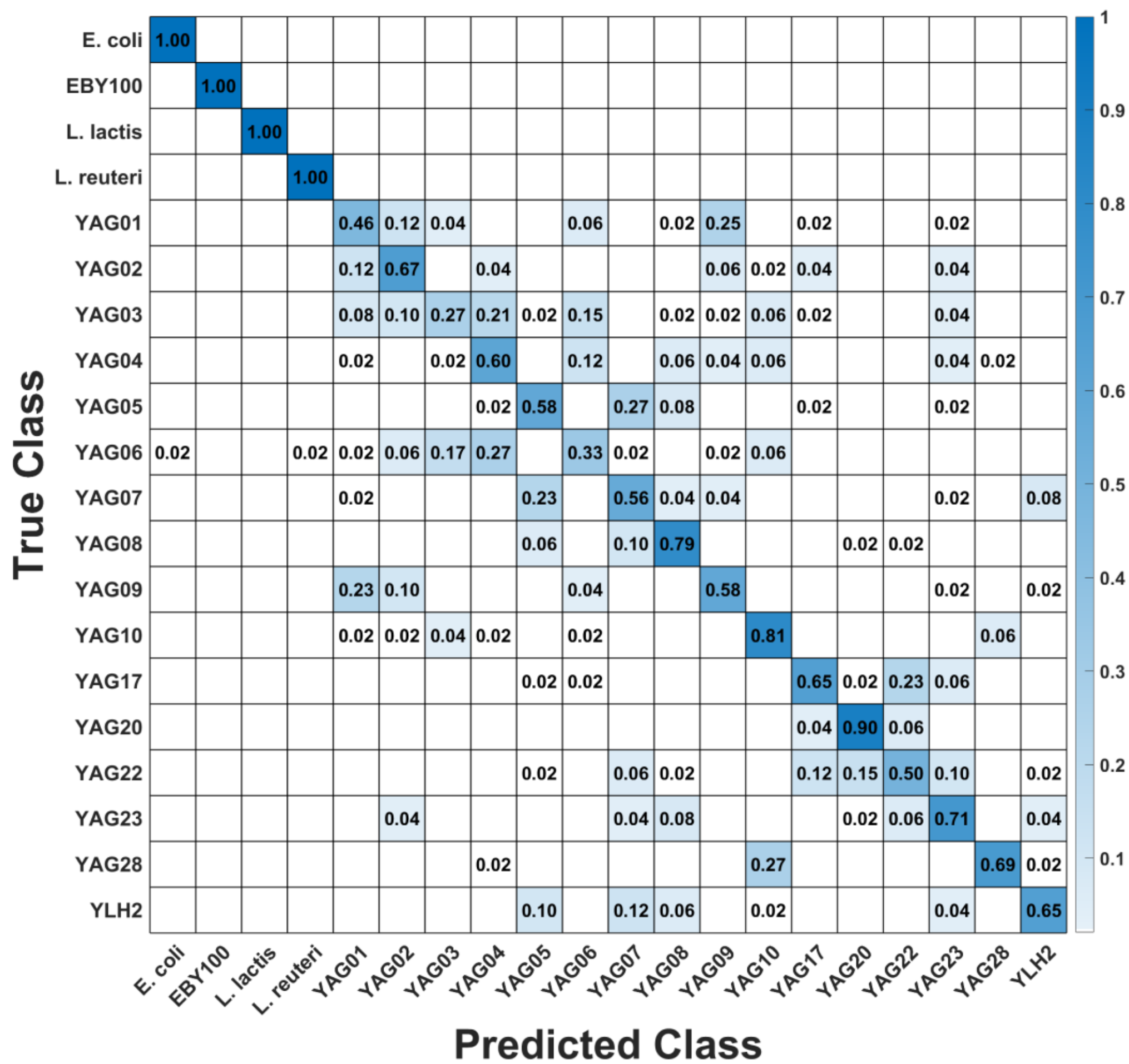

Figure S36 Row-wise normalized confusion matrix for multiclass classification of cells using a Convolutional Neural Network (Overall accuracy- 68.75%).

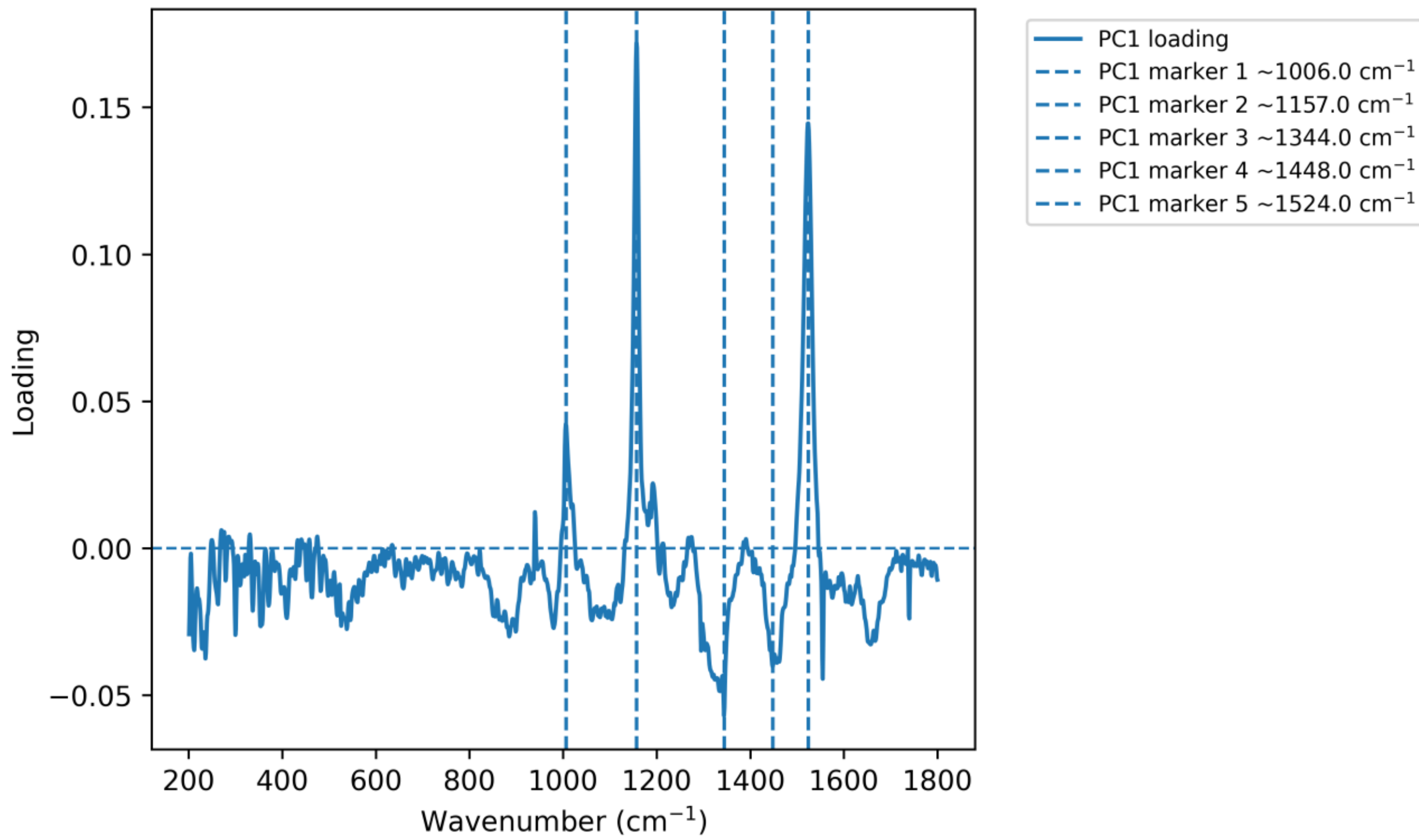

Figure S37 PCA loading plot highlighting the major wavenumbers contributing to PC1 for the cells dataset. The PC1 loading spectrum shows prominent contributing bands at approximately 1006, 1157, 1344, 1448, and 1524 cm^{-1}, marked by vertical dashed lines.

**Wavenumber (cm^{-1})**	**Vibration Mode**	**Metabolite**	**References**
~1157	C-C stretch	β-carotene, lycopene	[6]
~1449	CH_2 bend	fatty acids	[6]
~1524	C=C stretch	unsaturated fatty acids	[6]

Table S19 Raman peak assignments for mutated yeast strains.

REFERENCES

1. Lin-Vien, D., et al., *The handbook of infrared and Raman characteristic frequencies of organic molecules*. 1991: Elsevier.
2. Feng, X., et al., *Raman active components of skin cancer.* Biomedical Optics Express, 2017. **8**(6): p. 2835-2850.
3. Stiebing, C., et al., *Raman imaging of macrophages incubated with triglyceride-enriched oxLDL visualizes translocation of lipids between endocytic vesicles and lipid droplets.* Journal of Lipid Research, 2017. **58**(5): p. 876-883.
4. Rimskaya, E., et al., *Multi-Wavelength Raman Differentiation of Malignant Skin Neoplasms.* International Journal of Molecular Sciences, 2024. **25**(13): p. 7422.
5. Tsai, S.-L., et al., *Functional Assembly of Minicellulosomes on the <i>Saccharomyces cerevisiae</i> Cell Surface for Cellulose Hydrolysis and Ethanol Production.* Applied and Environmental Microbiology, 2009. **75**(19): p. 6087-6093.
6. Godara, A., et al., *Beneficial mutations for carotenoid production identified from laboratory-evolved Saccharomyces cerevisiae.* J Ind Microbiol Biotechnol, 2019. **46**(12): p. 1793-1804.
